# Tumor-associated MerTK promotes a pro-inflammatory microenvironment and enhances immune checkpoint inhibitor response in triple-negative breast cancer

**DOI:** 10.3389/fonc.2025.1579214

**Published:** 2025-05-05

**Authors:** Bridget E. Crossman, Regan L. Harmon, Mari Iida, Jillian M. Adams, Candie Y. Lin, Christine E. Glitchev, Terry D. Juang, Sheena C. Kerr, Roxana A. Alexandridis, Meredith Hyun, David T. Yang, Irene Kang, Ravi Salgia, Deric L. Wheeler

**Affiliations:** ^1^ Department of Human Oncology, University of Wisconsin, Madison, WI, United States; ^2^ Department of Biomedical Engineering, University of Wisconsin, Madison, WI, United States; ^3^ School of Medicine and Public Health, University of Wisconsin Carbone Cancer Center, University of Wisconsin, Madison, WI, United States; ^4^ Department of Biostatistics and Medical Informatics, University of Wisconsin, Madison, WI, United States; ^5^ Department of Pathology and Laboratory Medicine, University of Wisconsin, Madison, WI, United States; ^6^ Department of Medical Oncology and Therapeutics Research, City of Hope Comprehensive Cancer Center, Duarte, CA, United States

**Keywords:** MERTK, TNBC, PDL1, CTLA4, ICI, biomarker

## Abstract

**Introduction:**

Triple-negative breast cancer (TNBC) is an aggressive subtype of breast cancer with no targeted treatment modalities. Currently, combination chemotherapy and immune checkpoint inhibitor (ICI) therapy are options for many TNBC patients; however, their efficacy is limited. Understanding what makes TNBCs responsive to immune therapy is crucial for improving patient outcomes.

**Methods:**

We investigated the role of MerTK expression in TNBC using syngeneic and immunodeficient mouse models, human and murine cells lines, and human clinical samples. Flow cytometry, immunohistochemistry, RNA, multiplex ELISA, immunohistochemistry and multiplex immunofluorescence analysis were used to probe the effects of MerTK expression on the tumor immune microenvironment.

**Results:**

Overexpression of MerTK in TNBC syngeneic mouse models leads to a marked delay in tumor growth, coupled with significant increases in anti-tumor M1 macrophage, CD4+ T cell, active CD8+ T cell, active NK cell, and NKT cell populations. This increase in pro-inflammatory cells contrasted with decreased anti-inflammatory polymorphonuclear myeloid-derived suppressor cells (PMN-MDSCs) and regulatory T cells (Tregs) in the TIME. In addition, tumors overexpressing MerTK exhibited very high sensitivity to both aPDL1 and aCTLA4 therapies, leading to durable tumor control and, in some cases, complete tumor regression without recurrence. Further, using Vectra multispectral analysis, elevated MerTK expression in human clinical samples was associated with increased levels of pro-inflammatory immune cells. In vivo and human clinical data suggest that tumor-bound MerTK expression is independent of PD-L1 expression in TNBC.

**Conclusion:**

These preclinical findings indicate that MerTK could serve as an independent predictive biomarker for ICI response in TNBC, potentially expanding the cohort of late-stage TNBC patients eligible for ICI therapy while reducing toxicity in early-stage patients by treating only those predicted to respond.

## 1 Introduction

Breast cancer is the most common type of cancer and the fifth leading cause of cancer death in women worldwide, with 2.3 million new cases diagnosed and approximately 650,000 associated deaths in 2022 (1). Triple-negative breast cancer (TNBC) is a highly aggressive subtype of breast cancer, constituting 15-20% of breast cancer diagnoses, and is associated with younger age and higher stage disease at the time of diagnosis and African American race/ethnicity (2). TNBCs are characterized by their lack of expression of the estrogen receptor (ER), progesterone receptor (PR), and human epidermal growth factor receptor 2 (HER2), and thus are not candidates for existing therapies that target ER, PR, or HER2 in breast cancer (3). Treatment options for most TNBC patients include surgery, chemotherapy, and radiation, with some patients being candidates for immune checkpoint inhibitor therapy based on disease stage and PD-L1 status (4). Five-year survival rates for patients with regional and distant metastatic disease are low (66% and 14%, respectively), with a 50% recurrence rate, indicating a critical need for new treatment strategies and biomarkers for therapeutic response in these patient populations (5, 6).

The TAM (Tyro3, Axl, MerTK) family of receptor tyrosine kinases (RTKs) are well-known to play critical roles in cell survival, proliferation, adhesion, and migration, as well as regulating the release of cytokines (7). MerTK, the focus of this study, is typically expressed by immune cells (i.e., macrophages, dendritic cells, NK cells) but is overexpressed in many types of cancers, including TNBC (7–9). In solid tumors, MerTK expression correlates with increased signaling through Akt, mTOR, and MEK/Erk and has been implicated in promoting tumor cell migration and invasion (7, 8). Our group has previously demonstrated that overexpression of tumor-bound MerTK in TNBC promotes tumor progression and metastatic potential in *in vitro* and immunodeficient mouse models (9). In recent years, MerTK has emerged as a possible target for treating solid tumors, ushering in the use of small molecule inhibitors against the TAM family of RTKs in many cancer types (10–13). Further, the expression of various RTKs has been shown to have modulatory effects on the tumor immune microenvironment (TIME) that can result in therapeutic resistance (12, 14–17).

The TIME is a key factor in anti-tumor immunity and response to immune checkpoint therapy, referred to on a relative scale from “cold,” or immune-excluded, to “hot,” or highly immune infiltrated. Hotter tumors, such as melanoma and non-small cell lung cancer (NSCLC), respond well to immune checkpoint inhibitors (ICIs). In comparison, colder tumors, such as breast and head and neck squamous cell carcinoma (HNSCC), are often resistant to ICI therapy (18). While breast cancers are typically considered cold, the TNBC TIME, while highly heterogeneous, is thought to be hotter relative to hormone receptor-positive breast cancers (19). Pembrolizumab, an ICI targeting PD-1, is currently FDA-approved in combination with chemotherapy for early-stage TNBCs and late-stage TNBCs that express PD-L1; however, durable response rates remain low (20–22). While combination chemotherapy and ICI treatment is a viable option for some TNBC patients, what makes TNBCs sensitive and resistant to immune therapy is still unknown.

Given that TNBCs are relatively cold and MerTK is associated with tumor progression in TNBC, we initially hypothesized that tumor-bound MerTK promotes a cold TIME in TNBC, which may lead to resistance to ICIs. In contrast, we report in this study that tumor-bound MerTK plays a unique role in promoting a hot TIME in TNBC, and high expression of tumor MerTK sensitizes tumors to ICI therapy. Our data suggests that MerTK overexpression on the TNBC tumor leads to robust increases in anti-tumor immune infiltrate, cytotoxic lymphocyte activation, immune-mediated tumor killing, and remarkable tumor control in murine models, in some cases complete tumor regression without recurrence, when treated with ICI. Furthermore, we report that tumor-bound MerTK and PD-L1 are expressed independently. Though further validation is required, this preclinical study identifies tumor MerTK as a potential predictive biomarker to widen the cohort of patients eligible for ICI therapy.

## 2 Materials and methods

### 2.1 Cell lines

The mouse TNBC cell line EMT6 was purchased from American Type Culture Collection (ATCC, Manassas, VA, USA). The mouse 4T1 cell line was gifted from Dr. Caroline Alexander (Univ. of Wisconsin). The human breast cancer cell line SUM102 was obtained from Asterand (Detroit, MI, USA). Cell lines were authenticated by the respective source, and the SUM102 cell line was confirmed by short tandem repeat (STR) analysis. All cell lines were maintained in their respective culture media (Corning, Corning, NY, USA) with 10% fetal bovine serum (FBS) and 1% penicillin and streptomycin: 4T1 in Roswell Park Memorial Institute (RPMI)-1640 and EMT6 and SUM102 in Dulbecco’s Modified Eagle’s Medium (DMEM).

### 2.2 Plasmid constructs and transfection

pDONR223-MERTK was a gift from William Hahn & David Root (Addgene plasmid #23900; http://n2t.net/addgene:23900; RRID: Addgene 23900) and was subcloned into the pcDNA6.0 expression vector and transfected into SUM102 as previously described (9). pcDNA3.1-mouse MerTK was purchased from GenScript (Piscataway, NJ, USA). Transfection into 4T1 was performed using Lipofectamine 3000 and Opti-MEM (Life Technologies, Carlsbad, CA, USA) according to the manufacturer’s instructions. Antibiotic selection was started 48 hours after transfection with G418 (250ug/mL) in growth media. For the generation of MerTK tetracycline-inducible EMT6 clones, MerTK was subcloned into the pcDNA4/TO expression vector (Thermo Fisher Scientific, Waltham, MA, USA). Co-transfection with pcDNA6/TR (Thermo Fisher Scientific) into EMT6 was performed using Lipofectamine 3000 and Opti-MEM (Life Technologies) according to the manufacturer’s instructions. Antibiotic selection was started 48 hours after transfection with blasticidin (1 ug/mL) and Zeocin (50 ug/mL) (Thermo Fisher Scientific) in growth media.

### 2.3 Western blot analysis

Whole-cell lysis, protein quantification, and Western blot analysis were performed as previously described (23). Membranes were incubated overnight at 4C with the following primary antibodies: MerTK (1:1000, ab184086, Abcam, Cambridge, United Kingdom), MerTK (1:1000, CS4319, Cell Signaling Technologies, Danvers, MA, USA), PD-L1 (1:1000, 17952-1-AP, Proteintech, Rosemont, IL, USA), GAPDH (1:5000, CS2118, Cell Signaling Technologies).

### 2.4 Gas6 ELISA

Cells were plated at 30,000 cells/mL (EMT6 cells plated in the presence of 1ug/mL doxycycline) and supernatant was collected after 48 hours. The supernatant was centrifuged to remove debris and Gas6 levels were quantified by sandwich ELISA using the Mouse Gas6 ELISA Kit (#ELM-GAS6; RayBiotech, Peachtree Corners, GA, USA) according to manufacturer’s instructions.

### 2.5 Phosphorylated protein array

Cells were seeded 48 hours prior to performing the assay (EMT6 cells plated in the presence of 1ug/mL doxycycline), stimulated with 200ng/mL Gas6, and lysates were subsequently collected. Phospho-protein analysis was performed using the Human/Mouse MAPK Phosphorylation Array (#AAH-MAPK-1-2, RayBiotech) according to manufacturer’s instructions. Membranes were imaged on an Azure Biosystems C600 imager (Azure Biosystems, Dublin, CA). Relative spot intensities were calculated and normalized using the “Protein Array Analyzer Version 1.1.c” package for ImageJ.

### 2.6 Therapeutic antibodies

Therapeutic antibodies for *in vivo* use were obtained from BioXCell (Lebanon, NH): Mouse IgG1 isotype control (#BE0083), anti-mouse PD-L1 (#BP0101), and anti-mouse CTLA-4 (#BP0131).

### 2.7 *In vivo* tumor growth, ICI, and tetracycline-inducible studies

Animal procedures and maintenance were conducted in accordance with the University of Wisconsin-Madison School of Medicine and Public Health IACUC guidelines. Female BALB/c and NCG mice (4–6 weeks old) were obtained from Charles River Laboratories (Wilmington, MA, USA). Female athymic nude mice (4–6 weeks old) were obtained from Inotiv (Indianapolis, IN, USA). For tumor growth studies, 5x10^5^ cells were resuspended in PBS with Matrigel (50% v/v, R&D systems, #3433-005-01) and inoculated by subcutaneous injection bilaterally into the dorsal flank of each mouse. For tumor inoculation, mice were initially anesthetized with 5% isoflurane in 100% oxygen at a flow rate of 2L/min for induction, followed by maintenance at 2% isoflurane with an oxygen flow rate of 1L/min. Tumors were measured two times per week using a digital caliper. For ICI treatment studies, tumors were allowed to grow to ~50mm^3,^ and mice were randomized into groups before initiation of treatment. Either IgG or therapeutic antibody was administered by intraperitoneal injection. For the dox-inducible study, tumors were allowed to grow to ~100mm^3,^ and the diet was switched to 200mg/kg dox chow (Teklad, Madison, WI; #TD.0052).

### 2.8 Immunohistochemistry

The tissue microarray (TMA) of TNBC clinical samples was obtained from TissueArray.com LLC (#BR1301, Derwood, MD, USA), Translational Research Initiatives in Pathology (TRIP lab, Univ. of Wisconsin and stained according to the manufacturer’s recommendation. Syngeneic tumor tissue was fixed in 10% neutral buffered formalin, embedded in paraffin, and stained using either the Vectastain Universal Quick HRP Kit or the ImmPress HRP Goat Anti-Rat Kit (PK-8800 or MP-7444-15, Vector Laboratories, Newark, CA, USA). Antigen retrieval was performed using TRIS-EDTA buffer (pH 9.0) or Citrate buffer (pH 6.0) and incubated overnight at 4°C, with or without primary antibody as a negative staining control. Antibody binding was detected using 3,3’-diaminobenzidine substrates (SK-4105, Vector Laboratories) and counterstained with hematoxylin QS (H-3404, Vector Laboratories). Samples were examined using an Olympus BX51 microscope. Representative images are shown at a magnification of 20x. Quantitation of staining intensity was performed as previously described (24). **Supplementary Table S1** outlines antibodies used.

### 2.9 Immunofluorescence

Cells were plated in 4-well Millicell EZ chamber slides (Millipore Sigma, Burlington, MA, USA) 24h before staining. Cells were fixed in 3% formaldehyde, permeabilized in 0.3% Triton X-100, and blocked in normal goat serum. Cells were stained with MerTK primary antibody (sc-365499, Santa Cruz Biotechnologies, Dallas, TX, USA) overnight at 4C. Cells were then stained with a secondary antibody (goat anti-mouse A488, Invitrogen). Actin was stained using the ActinRed 555 ReadyProbes Reagent (Invitrogen), and nuclei were stained with DAPI. Slides were imaged on a Leica SP8 Confocal WLL STED Microscope. MerTK levels were quantified using FIJI V2.14.0/1.54f.

### 2.10 Tumor dissociation and flow cytometry

Tumors were harvested and dissociated as previously described (12). Briefly, mice were euthanized by CO_2_ asphyxiation followed by cervical dislocation and tumors were collected, minced, and dissociated using a gentleMACS Octo Dissociator with Heaters (Miltenyi Biotech, Bergisch Gladbach, North Rhine-Westphalia, Germany). Cells in suspension were stained with a live/dead dye, FC receptor binding inhibitor, and fluorescent antibodies. Samples were run on an Attune Nxt flow cytometer (Thermo Fisher Scientific), and analysis was performed in FlowJo (BD, Franklin Lakes, NJ; RRID: SCR_008520). **Supplementary Table S2** outlines the fluorescent antibodies used, and **Supplementary Table S3** outlines gating paths.

### 2.11 Multiplex immunofluorescence

The TNBC clinical sample TMA was obtained from Tissuearray.com LLC (#BR1301) and multiplex immunofluorescent staining with the Opal system was performed by the Translational Research Initiatives in Pathology (TRIP lab, Univ. of Wisconsin) according to the manufacturer’s instructions (Akoya Biosciences, Boston, MA, USA). **Supplementary Table S4** outlines antibodies used for staining and corresponding Opal secondary for the immune profiling study. **Supplementary Table S5** outlines antibodies used for the MerTK vs. PD-L1 correlation study. Nuclei were counterstained with DAPI. The stained TMA slide was scanned on the Vectra 2 (Akoya Biosciences) at 4x magnification. Nine images were stitched together to generate a full field of view for each core on the TMA. The scanned images were analyzed using the InForm v.2.4.8 software (Akoya Biosciences). A spectral library was utilized to unmix signals from each analyte, and analytes were pseudocolored as outlined in **Supplementary Tables S4**, **S5**. Tissues were segmented into tumor and stroma, and cells were segmented into nucleus, membrane, and cytoplasm compartments in InForm. Analytes were quantified as either optical density (OD) or percent of segment area positive for each analyte.

### 2.12 PBMC migration assay

Cell spheroids were prepared by combining 20% methylcellulose and cells in culture media and pipetting droplets on an untreated Nunc OmniTray (Thermo Fisher Scientific) and incubated for 24 hours. KOALA microfluidic devices were fabricated as previously described (25). Channels on the KOALA device were coated with 50μg/ml P-selectin in PBS at 4C overnight. Channels were then washed and filled with 4μl of RPMI + 10% FBS + Hoechst (1:1000) containing isolated human PBMCs at 625 cells/μl and incubated for ten minutes at room temperature. The spheroids were harvested, and rat tail collagen I (Corning) was added to each spheroid at a final concentration of 3.6mg/ml diluted in tumor cell culture media. The spheroids in collagen were added to the KOALA lid, and the collagen was allowed to polymerize for 30–60 minutes. The base of the KOALA devices containing the PBMCs was placed onto an H101-2xGS-M holder (Okolab, Pozzuoli, Napoli, Italy) on a Nikon TiE1 microscope (Nikon Instruments, Melville, NY, USA) with an incubated stage set to 37C with oxygen and humidity control. The KOALA lid was lowered onto the slide, and all conditions were imaged at 5-minute intervals at 20x magnification over 3 hours. Images were analyzed using FIJI and the TrackMate plugin to track cell migration (26). Forward Migration Index (FMI) and Center of Mass (COM) were analyzed using the IBIDI Chemotaxis and Migration Tool (IBIDI, Gräfelfing, Germany).

### 2.13 RNA isolation, cDNA synthesis, and qPCR

Total RNA from cultured cells was isolated using the RNeasy Mini kit (Qiagen, Germantown, MD, USA) according to the manufacturer’s recommendation. After quantification of RNA by Nano-drop spectrophotometer (Thermo Fisher Scientific), cDNA synthesis was performed using the qScript cDNA Supermix (Quantbio, Beverly, MA, USA). Taqman Advanced Master Mix and Taqman probes (MerTK Hs1031973, Mm00434920; Actin Mm00607939; 18S 433760; Thermo Fisher Scientific) were used, and quantitative real-time PCR (qPCR) was performed using the QuantStudio 6 Pro (Thermo Fisher Scientific). Gene expression was analyzed using the ΔΔ*C_T_* method. Actin and 18S were used as normalization controls.

### 2.14 Nanostring nCounter analysis

Total RNA from tumors was isolated using the RNeasy Mini kit (Qiagen) according to the manufacturer’s recommendation. After quantification of RNA by Nano-drop spectrophotometer (Thermo Fisher Scientific), RNA quality was assessed by the UW-Madison Gene Expression Center. The NanoString nCounter PanCancer IO 360 panel (NanoString Technologies, Seattle, WA) was used to analyze RNA expression levels. The UW Madison Translational Research Initiatives in Pathology (TRIP) lab performed sample preparation, hybridization, and scanning on the nCounter MAX digital analyzer according to manufacturers instructions. Quality control was performed, and normalized counts were obtained from raw count data of n=4 samples per group using nSolver software (NanoString Technologies).

### 2.15 Multiplex cytokine and chemokine analysis

Cytokine and chemokine levels from cell culture supernatant were measured using the ProcartaPlex Mouse Cytokine & Chemokine Convenience Panel (Thermo Fisher Scientific, EPXR360-26092-901) according to manufacturer’s instructions. Samples were read on a Luminex xMAP INTELLIFLEX System (Thermo Fisher Scientific) and analyzed using the accompanying ProcartaPlex Analysis App (Thermo Fisher Scientific).

### 2.16 Statistical analysis

For all statistical tests, differences were considered significant when *P*<0.05. Analysis of flow cytometry, immunohistochemistry, immunofluorescence, Luminex, NanoString and PBMC migration studies was performed using GraphPad Prism (GraphPad Software, Inc; RRID: SCR_002798). Independent two-sided t-tests were used when comparing means between two groups, while one-way ANOVA with Dunnett’s tests were used to compare three or more groups.

Analysis of tumor growth rates was carried out in R 4.4.1. In general, linear mixed models accounting for mouse ID using a random intercept were fit on tumor volume with treatment, day, and the interaction between treatment and day included as fixed effects. If tumor volumes did not contain any values of zero, the outcome was log-transformed to satisfy model assumptions. Otherwise, tumor volumes were transformed by log base 10 after adding half the minimum threshold (log(Y+(minY/2))) to handle the presence of zeroes. In a few cases however, transformation was not sufficiently normal to meet assumptions of the linear mixed model. These exceptions included NCG mice injected with 4T1 MerTK & EMT6 MerTK overexpressing clones, and BALB/c mice with 4T1 MerTK clones, for which analogous GEE models with exchangeable correlation were used instead. For all models, contrasts between specific treatment group pairs were adjusted for multiple comparisons using Sidak’s method.

## 3 Results

### 3.1 TNBCs overexpressing MerTK exhibit delayed tumor growth *in vivo*

**Figure 1:**
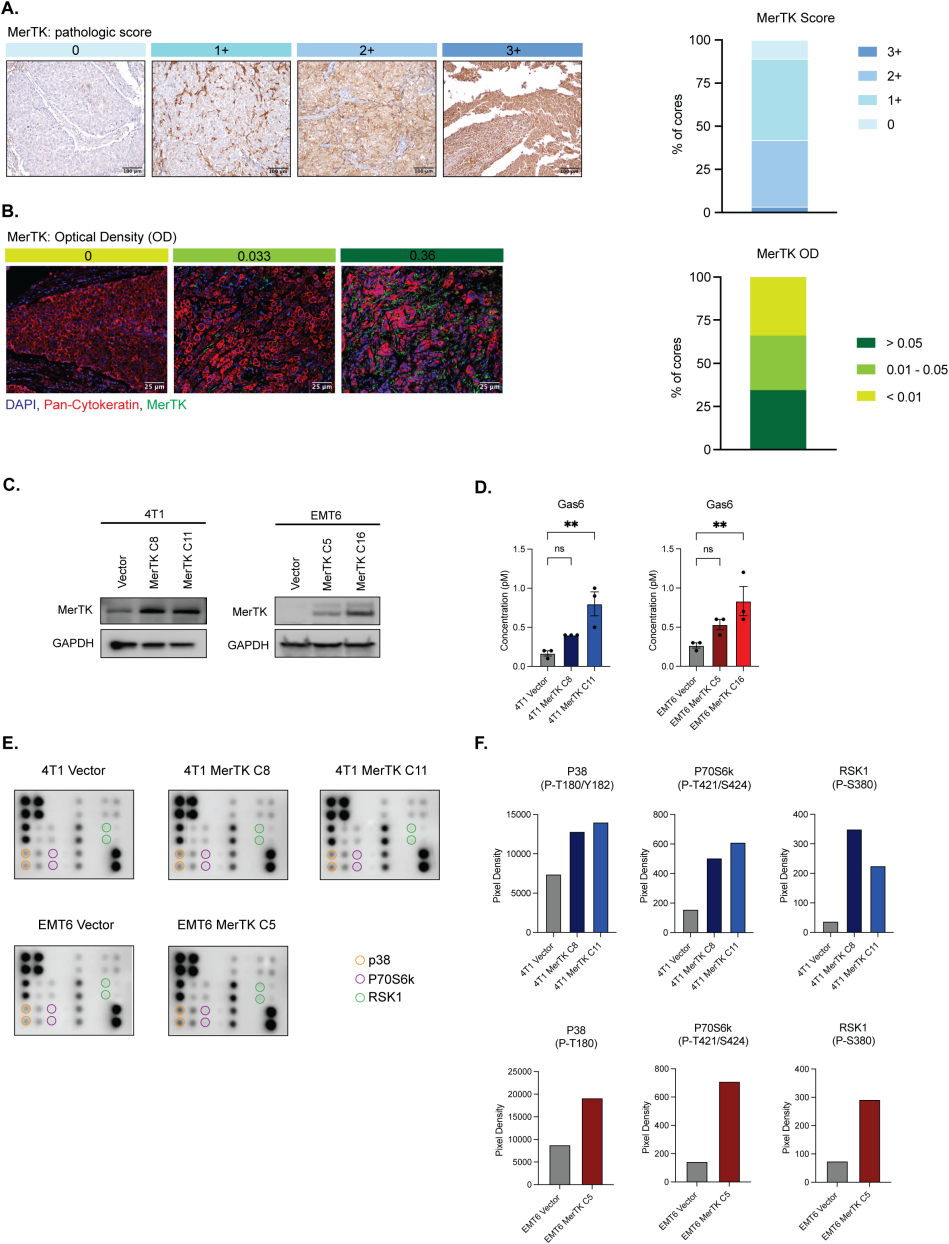
TNBC clinical samples express MerTK and the creation of a model of MerTK-overexpressing mouse TNBC cell lines. **(A)** MerTK is differentially expressed in human TNBC clinical samples as analyzed by IHC (n=137 patients). Pathological score was determined (D.T.Y) on a scale of 0-3+, and representative images for each score are shown. **(B)** MerTK is differentially expressed in human TNBC clinical samples as analyzed by IF (n=119 patients). Optical density (OD) was calculated using the inForm software. Representative images for each OD range are shown. **(C)** Whole cell lysate was harvested from 4T1 MerTK and EMT6 MerTK clones and their accompanying vector controls. MerTK expression was determined by immunoblot. GAPDH is shown as a loading control. **(D)** Cell culture supernatant was collected from confluent plates of 4T1 MerTK and EMT6 MerTK clones and their accompanying vector controls. Secreted Gas6 levels were analyzed by ELISA. **(E)** Lysates from 4T1 and EMT6 MerTK clones and their accompanying vector controls were evaluated for phosphorylation of downstream effectors of MerTK by phospho-protein array. **(F)** Pixel density of phosphorylated proteins circled in **(E)**. Mean values are shown (n=2). ** P<0.01; ns, not signficant.

Our lab previously showed that MerTK is overexpressed in human TNBC cell lines and PDX models (9, 27). To further characterize MerTK expression in human TNBCs, we analyzed a TNBC TMA for MerTK expression by immunohistochemical (IHC) and immunofluorescent (IF) staining. MerTK was found to be highly expressed (pathologic score >2+ as scored by a board-certified pathologist (D.T.Y) in about 30% (45/137) of human TNBCs by IHC (**Figure 1A**) and highly expressed (optical density >0.05) in 34% (40/119) of human TNBCs by IF (**Figure 1B**). These results revealed that about 30% of TNBC patients have relatively high MerTK expression. Previous work from our lab has also indicated that TNBC cells overexpressing MerTK exhibit higher levels of proliferation in immune-compromised mice and metastatic potential *in vitro* (9). Given that MerTK is highly expressed in about one-third of human TNBCs and MerTK overexpression is linked to tumor progression *in vitro*, we hypothesized that tumors overexpressing MerTK would grow faster *in vivo* and lead to an immunosuppressive TIME. To test this hypothesis, we generated MerTK-overexpressing clones in two independent murine models of TNBC [4T1 (28) and EMT6 (29)] with low endogenous levels of MerTK. We generated one vector control and two independent, stable MerTK-overexpressing clones in the 4T1 model (4T1 Vector, 4T1 MerTK C8, and 4T1 MerTK C11). Additionally, we generated a doxycycline (dox)-inducible model of MerTK overexpression in EMT6 (EMT6 Vector, EMT6 MerTK C5, and EMT6 MerTK C16). Stable or induced MerTK overexpression was confirmed by Western blot (**Figure 1C**), quantitative real-time PCR (qPCR, **Supplementary Figure S1A**), flow cytometry (**Supplementary Figure S1B**) and immunofluorescence (IF, **Supplementary Figures S1C, D**). Further, it is known that cells expressing MerTK secrete Gas6, the ligand required for MerTK activation (30). Here we demonstrate that our MerTK-overexpressing cell model does secrete Gas6 (**Figure 1D**), and a phospho-protein array demonstrates activation of downstream effectors of MerTK (**Figures 1E, F**).

**Figure 2:**
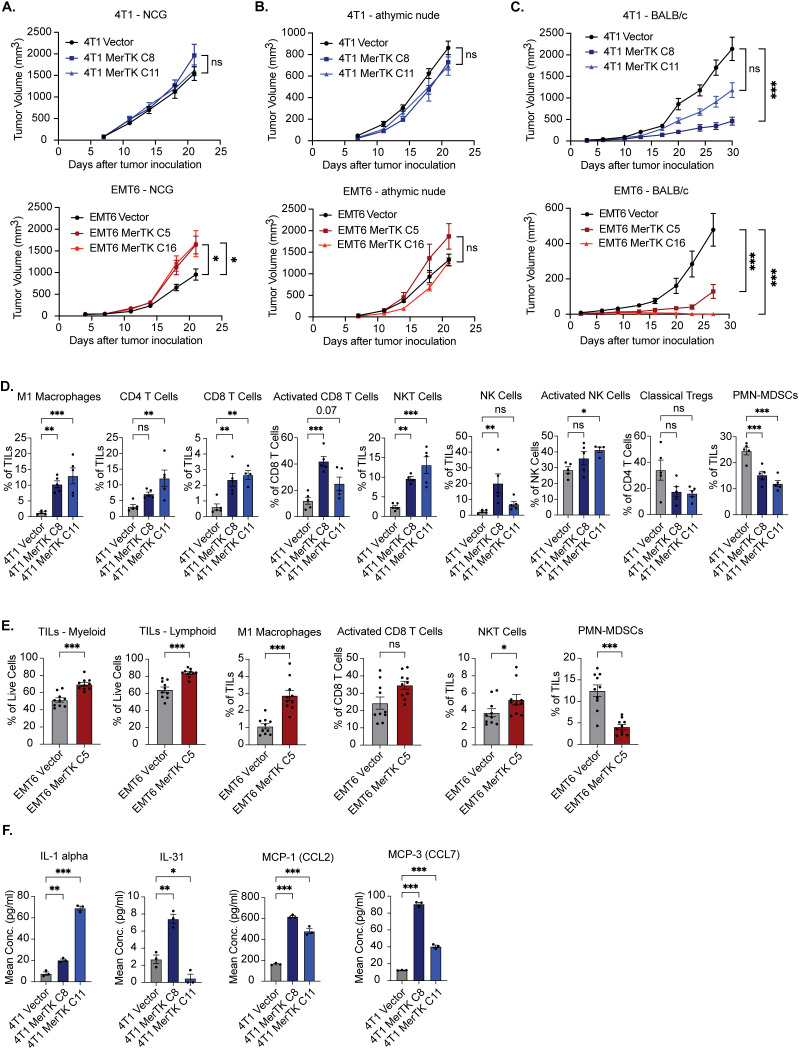
MerTK expression promotes delayed tumor growth and a hotter TIME in syngeneic models of TNBC. **(A)** 4T1 MerTK and EMT6 MerTK overexpressing clones and their vector controls were injected onto the flanks of NCG mice, and tumor volume was measured twice weekly (n=8 tumors per group). **(B)** 4T1 MerTK and EMT6 MerTK overexpressing clones and their vector controls were injected onto the flanks of athymic nude mice, and tumor volume was measured twice weekly (n=8 tumors per group). **(C)** 4T1 MerTK and EMT6 MerTK overexpressing clones and their vector controls were injected onto the flanks of BALB/c mice, and tumor volume was measured twice weekly (n=6–16 tumors per group). Mice bearing EMT6 tumors were fed dox-containing chow (equivalent to 200mg/kg/day) beginning one week prior to tumor inoculation. Mean values and SEMs are shown. **(D, E)** Tumor-infiltrating lymphocytes (TILs) were analyzed via flow cytometry (**D**: 4T1, **E**: EMT6). Mean values and SEMs are shown (n=5–10 tumors per group). **(F)** Cytokines and chemokines released by tumor cells into cell culture media were analyzed via Luminex. Mean values and SEMs are shown. (n = 3 per group). *P<0.05; ** P< 0.01; ***P<0.001; ns, not significant.

To evaluate the effect of MerTK overexpression on tumor growth *in vivo*, 4T1 and EMT6 Vector and MerTK clones were inoculated bilaterally onto the flanks of NCG, athymic nude, and syngeneic (BALB/c) mice (n=8–16 tumors per group) by subcutaneous injection and tumor growth was measured over 21–30 days. Mice bearing EMT6 tumors were started on 200mg/kg dox chow 7 days prior to tumor inoculation and continued throughout the study to induce tumor MerTK expression. MerTK overexpression was associated with either unchanged or accelerated tumor growth in NCG mice (**Figure 2A**), unchanged tumor growth in athymic nude mice (**Figure 2B**), and significantly delayed tumor growth in BALB/c mice (**Figure 2C**) compared to vector control for both 4T1 and EMT6 tumor models. Moreover, EMT6 MerTK C16 tumors failed to establish in the syngeneic mouse model. Results from the tumor growth study in BALB/c mice for both the 4T1 and EMT6 tumor models were unexpected. Our data showing unchanged or accelerated growth of MerTK-overexpressing tumors in triple immunodeficient NCG mice lacking T cells, B cells, and natural killer (NK) cells and harboring low levels of macrophages and dendritic cells highlights the role of MerTK in promoting tumor growth. However, the striking abrogation of tumor growth observed in immune-competent mice suggests that overexpressing MerTK may modulate the tumor immune microenvironment (TIME) in TNBC.

### 3.2 MerTK overexpression promotes a pro-inflammatory TIME in two murine models of TNBC

To investigate whether the delay in MerTK-overexpressing tumor growth observed in BALB/c mice is immune-mediated, we performed flow cytometry using 4T1 Vector, MerTK C8 and MerTK C11, and EMT6 Vector and MerTK C5 tumors for immune profiling at endpoint (day 27 or day 30 post-inoculation, respectively) (**Figures 2D, E**). MerTK overexpression in 4T1 and EMT6 tumors was associated with strong anti-tumor immune infiltration. Analysis of 4T1 tumors revealed significant increases in anti-tumor M1 Macrophage, CD4+ T cell, CD8+ T cell, NK cell, and NKT cell populations, significant increases in CD8+ T cell and NK cell activation, and a significant decrease or trending decrease in pro-tumor polymorphonuclear myeloid-derived suppressor cell (PMN-MDSC) and regulatory T cell (Treg) populations respectively in MerTK overexpressing tumors compared to Vector control (**Figure 2D**, **Supplementary Figure S2**). Moreover, results from IHC analysis confirmed MerTK expression levels in tumors and a trending increase in proliferation as analyzed by Ki67 staining (**Supplementary Figure S3**). Additionally, IHC analysis corroborated the flow cytometry findings and showed increases in staining of CD8+ and CD4+ T cells, NK cells (NKp46), and macrophages (F4/80) in MerTK-overexpressing tumor sections relative to Vector control (**Supplementary Figure S3**). Similarly, analysis of EMT6 tumors revealed significant increases in total levels of tumor-infiltrating leukocytes (TILs) of both myeloid and lymphoid lineage, M1 macrophages, NKT cells, a trending increase in activated CD8+ T cells, and a significant decrease in pro-tumor PMN-MDSCs for EMT6 MerTK C5 tumors compared to Vector control (**Figure 2E**). Multiplex bead-based ELISA (Luminex) analysis of cell culture supernatant from 4T1 Vector, 4T1 MerTK C8, and 4T1 MerTK C11 tumor cells revealed significantly increased release of the pro-inflammatory cytokines IL-1 alpha and IL-31, and chemokines MCP-1 (CCL2) and MCP-3 (CCL7) (**Figure 2F**). Further, analysis of RNA isolated from cultured 4T1 Vector, 4T1 MerTK C8 and 4T1 MerTK C11 cells revealed genetic upregulation of pathways involved in JAK/STAT, PI3K/AKT, NFκB, TGFβ, and interferon signaling (**Supplementary Figure S4**). These data indicate that tumors overexpressing MerTK have high levels of anti-tumor immune infiltrate compared to vector control tumors, which may be responsible for the observed delay in tumor growth in syngeneic models.

### 3.3 Inducing MerTK expression correlates with a hotter TIME in the EMT6 model

**Figure 3:**
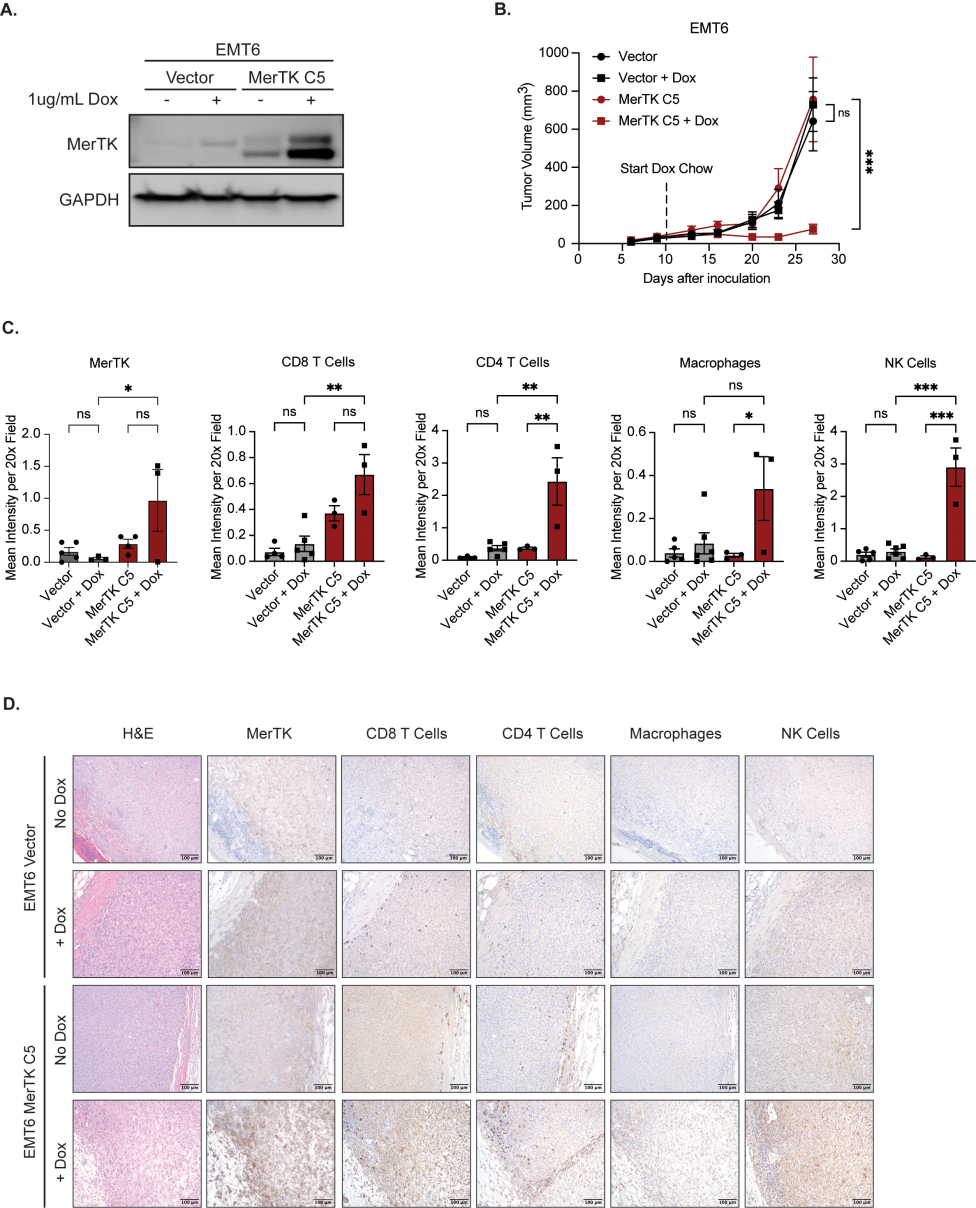
Inducing MerTK expression impairs tumor growth and promotes anti-tumor immune infiltration. **(A)** Vector and MerTK-overexpressing EMT6 cells were treated for 18 hours with vehicle or doxycycline (dox, 1ug/mL) prior to collecting whole-cell lysate. Lysates were immunoblotted for MerTK and GAPDH as a loading control. **(B)** Vector and MerTK-overexpressing EMT6 cells were inoculated onto the flanks of BALB/c mice. Tumor-bearing mice were treated with vehicle or doxycycline via chow (about 200mg/kg/day) starting at day 10 post-tumor inoculation, and tumor volume was measured twice weekly. Mean values and SEMs are shown (n=4–8 tumors per group). **(C)** Tumors were harvested and stained using IHC. Representative images are shown at 20x magnification. **(D)** Tumor immune infiltrate by IHC was quantified using FIJI V2.14.0/1.54f. Mean values and SEMs are shown (n=3–6 tumors per group). *P<0.05; ** P< 0.01; ***P<0.001; ns, not significant.

To further explore the link between MerTK expression and anti-tumor immune infiltrate, we utilized an inducible model of MerTK overexpression in the EMT6 model. In this system, MerTK expression is minimal in the absence of dox and elevated after 18-hour treatment with 1ug/mL dox added to cell culture media (**Figure 3A**). We chose the EMT6 MerTK C5 clone to carry out experimentation on due to the robust increase in MerTK expression following the addition of dox and its ability to establish a tumor in syngeneic mice. To test whether inducing MerTK expression can delay tumor growth and switch the TIME from anti-inflammatory to pro-inflammatory, EMT6 Vector and EMT6 MerTK C5 cells were inoculated bilaterally via subcutaneous injection on the flank of BALB/c mice (n=4–8 tumors per group) and tumors were measured twice weekly until they reached an approximate volume of 50mm^3^. On day 11 post-inoculation, half of the EMT6 Vector and half of the EMT6 MerTK C5 mice were kept on their regular diet while the other half of each group was switched to a dox-containing chow (approximately 200mg/kg/day). The chow was refreshed once per week, and measurements continued twice weekly until day 27 post-inoculation (**Figure 3B**). Results from this experiment show similar growth of all tumors before adding dox (day 0 to day 10). We observed no difference in growth pattern for EMT6 Vector tumors on mice fed a regular diet or dox chow. EMT6 MerTK C5 tumors on mice fed a regular diet with no induction of MerTK expression exhibited a similar growth pattern to both EMT6 Vector groups (Vector only and Vector + dox). However, EMT6 MerTK C5 tumors on mice fed dox chow to induce MerTK expression exhibited significant and strikingly delayed tumor growth starting at day 23 post-inoculation (12 days on dox) compared to both EMT6 Vector + dox and EMT6 MerTK C5 mice fed a regular diet. Tumors were harvested at the endpoint and evaluated by IHC for immune infiltration and MerTK expression. Significantly increased staining of CD8+ T cells, CD4+ T cells, macrophages (F4/80), and NK cells (NKp46) in EMT6 MerTK C5 + dox tumor sections was observed compared to EMT6 Vector + dox and EMT6 MerTK C5 fed regular diet (**Figures 3C, D**). These results suggest a direct role for MerTK in promoting delayed tumor growth by creating a pro-inflammatory TIME.

### 3.4 Overexpression of MerTK sensitizes 4T1 tumors to immune checkpoint inhibition

**Figure 4:**
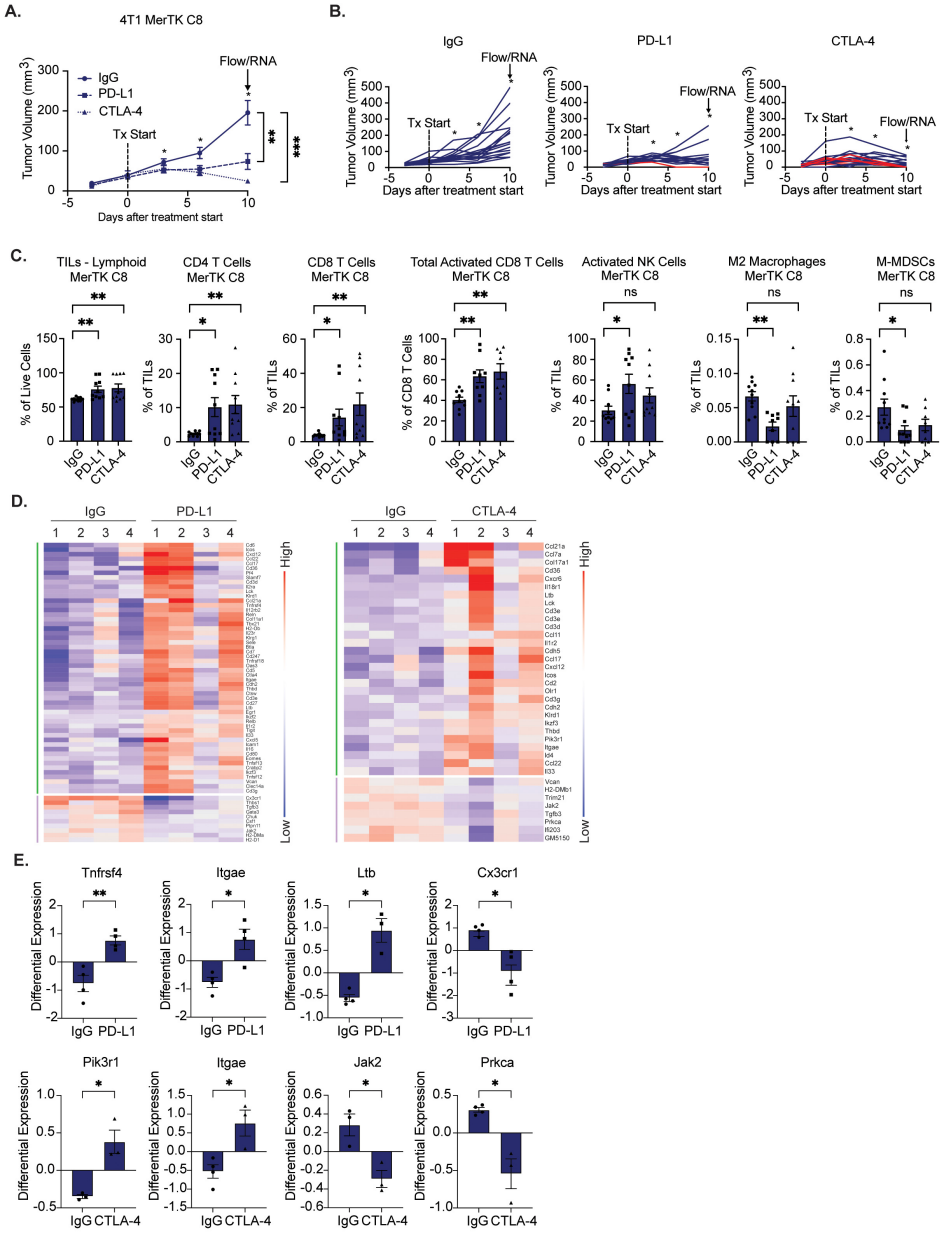
Tumors overexpressing MerTK are sensitive to immune checkpoint inhibition. A) 4T1 tumors overexpressing MerTK were inoculated onto the flanks of BALB/c mice. Tumor-bearing mice were treated with either IgG, αPD-L1 (15mg/kg), or αCTLA-4 (5mg/kg) therapeutic antibody three to four times, three days apart. Mean values and SEMs are shown (n=14–18 tumors per group). **(B)** Spaghetti plots for tumors presented in **(A)** Red lines indicate subjects whose tumors regressed without recurrence. **(C, D)** Immune profiling of 4–10 tumors in each group that were harvested three days after the third dose of antibody. **(C)** Tumor-infiltrating lymphocytes (TILs) were analyzed by flow cytometry. Mean values and SEMs are shown (8–10 tumors per group). **(D)** Heatmap of significantly altered genes involved in anti-tumor immunity for IgG control, PD-L1, and CTLA-4 treatment groups, clustered by upregulated (green) or downregulated (purple). Red and blue represent upregulation and downregulation, respectively. **(E)** Tumors were analyzed for gene expression by NanoString nCounter. Mean values and SEMs are shown (4 tumors per group). *P<0.05; ** *P*< 0.01; ***P<0.001; ns, not significant.

One key challenge in treating TNBCs is their intrinsic resistance to immune checkpoint therapy; only a small population of patients exhibit durable responses to pembrolizumab. Previous studies in melanoma and NSCLC suggest that tumors with higher levels of immune infiltrate are more sensitive to immune checkpoint inhibition (18). Due to the ability of MerTK to create a pro-inflammatory TIME, we hypothesized that tumors overexpressing MerTK would have increased sensitivity to ICI. To test this, we inoculated 4T1 MerTK C8 cells bilaterally onto the flanks of BALB/c mice by subcutaneous injection. Three days post-tumor inoculation, mice were treated with IgG control, αPD-L1 (15mg/kg), or αCTLA-4 (5mg/kg) by intraperitoneal injection three times (early timepoint) or four times (late timepoint) over 10 days (n=14–18 tumors per group). MerTK-overexpressing tumors exhibited robust sensitivity to both agents (**Figures 4A, B**). Ten tumors from each group were collected for immune profiling four days after receiving the third dose of antibody (early timepoint), and 6–10 tumors were subjected to four doses of antibody and observed for up to 120 days (late timepoint). Remarkably, of the late timepoint tumors, 16% (1/6) from the αPD-L1 and 90% (9/10) from the αCTLA-4 groups exhibited complete tumor regression without recurrence (**Figure 4B**). We observed infiltration of CD4+ and CD8+ T cells, increased CD8+ T cell and NK cell activation, and exclusion of pro-tumor M2 macrophages and mononuclear myeloid-derived suppressor cells (M-MDSCs) in tumors treated with therapeutic antibody as analyzed by flow cytometry (**Figure 4C**). At the RNA level, NanoString nCounter analysis revealed significant upregulation of genes associated with tumor immune infiltration, activity, and cytotoxicity (**Figure 4D**). Specifically, in PD-L1-treated tumors, Tnfrsf4, Itgae, and Ltb were significantly upregulated, and Cx3cr1 was significantly downregulated. In CTLA-4 treated tumors, Pik3r1 and Itgae were significantly upregulated, while Jak2 and Prkca were significantly downregulated compared to IgG control (**Figure 4E**). These results further indicate anti-tumor immune infiltration, retention, and activation in MerTK-overexpressing tumors after ICI treatment.

**Figure 5:**
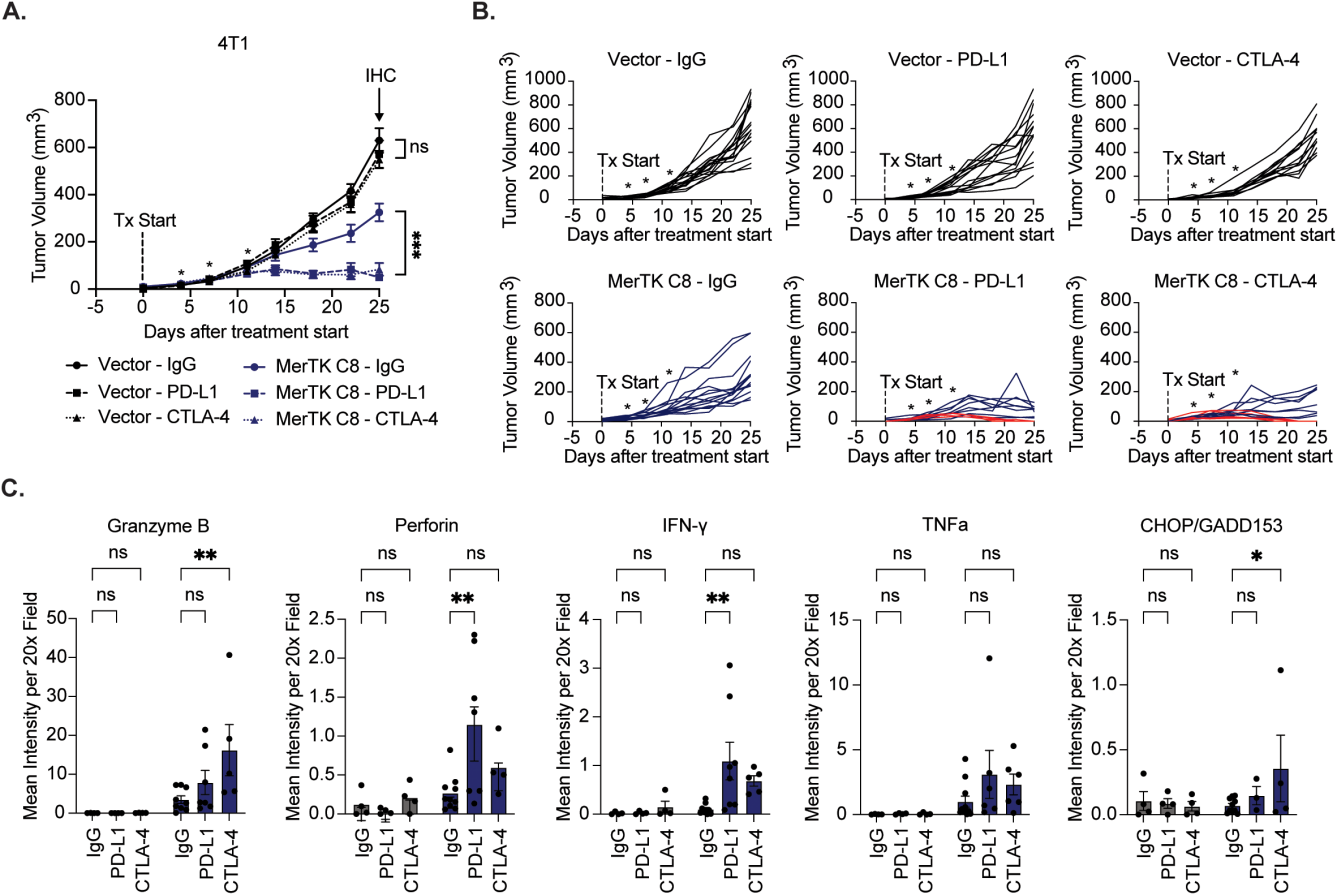
MerTK-negative tumors are resistant to immune checkpoint inhibition. **(A)** 4T1 vector and MerTK-overexpressing tumors were inoculated onto the flanks of BALB/c mice. Tumor-bearing mice were treated with IgG, αPD-L1 (15mg/kg), or αCTLA-4 (5mg/kg) therapeutic antibody four times 3–4 days apart. Mean values and SEMs are shown (n=12–16 tumors). **(B)** Spaghetti plots for each treatment group. Red lines indicate subjects whose tumors regressed without recurrence. **(C)** Cytotoxic granules (Granzyme B and Perforin), anti-tumor cytokines (IFN-γ and TNFα), and the apoptotic marker (CHOP/GADD153) were detected in tumor sections using IHC. Quantification was performed using FIJI V2.14.0/1.54f. Mean values and SEMs are shown (n=4–9 tumors per group). *P<0.05; ** *P*< 0.01; ***P<0.001; ns, not significant.

To assess ICI efficacy in a MerTK-negative model and further probe anti-tumor immune activity at the endpoint, we repeated the study and added the 4T1 Vector group. Either 4T1 Vector or 4T1 MerTK C8 cells were injected bilaterally onto the flanks of BALB/c mice by subcutaneous injection. Two days after inoculation, mice were treated with IgG control, αPD-L1 (15mg/kg), or αCTLA-4 (5mg/kg) by intraperitoneal injection four times over 10 days (n=12–16 tumors per group). All tumors were allowed to grow until the experimental endpoint 25 days after starting treatment, and tumors were assessed for immune-mediated cytotoxicity by IHC. 4T1 vector tumors showed no sensitivity to either ICI, while robust and durable tumor control was again observed in 4T1 MerTK C8 groups receiving therapeutic antibodies (**Figures 5A, B**). 33% (4/12) of 4T1 MerTK C8 tumors treated with αPD-L1 and 43% (6/14) of 4T1 MerTK C8 tumors treated with αCTLA-4 regressed without recurring over 120 days of observation. Furthermore, increased levels of cytotoxic granules (Granzyme B and Perforin), anti-tumor cytokines (IFN-γ and TNFα), and the apoptotic marker CHOP/GADD153 were observed in MerTK-overexpressing tumors in both ICI treatment groups (**Figure 5C**, **Supplementary Figure S5**) by IHC. Together, these data indicate that unlike MerTK-negative 4T1 Vector tumors, MerTK-overexpressing TNBCs are highly sensitive to treatment with either αPD-L1 or αCTLA-4 therapeutic antibodies, demonstrated not only by complete and durable response in up to 90% of cases, but increased anti-tumor immune infiltration, heightened activation of effector cells, and evidence that the infiltrating effector cells are exhibiting anti-tumor responses.

### 3.5 MerTK expression contributes to a hotter TIME in human TNBCs

**Figure 6:**
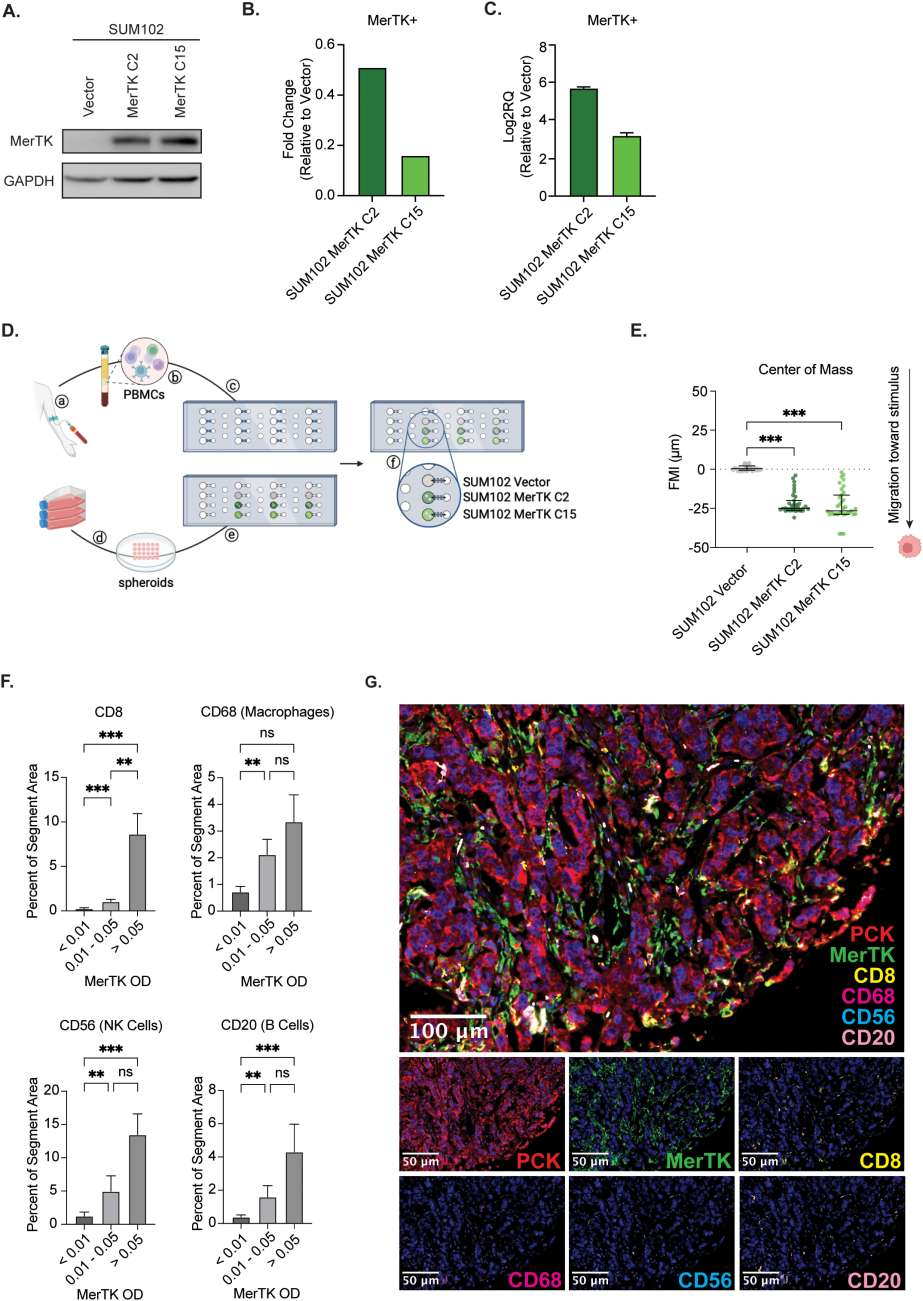
Human TNBCs expressing MerTK on the tumor exhibit increased lymphocyte recruitment. **(A)** Whole cell lysate was collected from SUM102 MerTK overexpressing clones and the vector control. Immunoblot analysis was performed for MerTK and GAPDH as a loading control. **(B)** MerTK expression was evaluated in SUM102 MerTK overexpressing clones and the vector control by flow cytometry. Fold changes relative to Vector control are shown (n=3 per group). **(C)** MerTK RNA expression was evaluated in SUM102 MerTK overexpressing clones and the vector control by qPCR. Fold changes relative to Vector control are shown (n=3 per group). **(D)** depiction of the KOALA assay workflow: (a) collection of human blood, (b) isolation of PBMCs, and (c) loading of PBMCs into lumens on the device base. (d) cultured cells were grown as spheroids and (e) embedded in the device lid. (f) The device lid is placed on the base, and the material flows through the lumen through capillary action. Created in BioRender. Wheeler, D. (2024) https://BioRender.com/f50n065. **(E)** PBMC forward migration index (FMI) was evaluated for each spheroid type. Data points represent the horizontal center of mass, where negative FMI indicates the migration of PBMCs toward a stimulus. Mean values and SEMs are shown (n=33–35 cells per group). **(F)** Human clinical TNBC TMA samples were evaluated for tumor immune infiltration by multiplex IF. Representative images are shown. **(G)** Tumor immune infiltrate was quantified using inform software. Mean values and SEMs are shown (n=36–41 samples per group). ** P<0.01; *** p<0.001; ns, not significant.

While the murine data presented suggests that MerTK expression may contribute to a pro-inflammatory TIME and sensitivity to ICIs in TNBC, it is critical to understand the role of MerTK in human TNBCs. To investigate this, we utilized a human model of TNBC overexpressing MerTK (SUM102 Vector, SUM102 MerTK C2, and SUM102 MerTK C15), confirmed by Western blot (**Figure 6A**), flow cytometry (**Figure 6B**) and qPCR (**Figure 6C**) in a microfluidic device to observe migration of primary blood mononuclear cells (PBMCs) in the presence or absence of MerTK. SUM102 Vector, SUM102 MerTK C2, or SUM102 MerTK C15 spheroids were generated for use in the kit-on-a-lid assay (KOALA) for PBMC migration (31). Cell spheroids were embedded into the lid of the device, while primary human PBMCs were loaded into a microfluidic channel in the base of the device. When the lid is placed on the base, flow between the lid and base is initiated by capillary action (**Figure 6D**). Directional flow of Hoechst-labeled PBMCs was observed by fluorescence microscopy and quantified by forward migration index (FMI; μm) of the center of mass of PBMCs. In a well with no spheroid implanted or SUM102 Vector spheroids implanted, no directional migration of PBMCs was observed. In the SUM102 MerTK C2 and MerTK C15 wells, however, PBMCs showed significant migration toward the MerTK-expressing cell spheroids (**Figure 6E**). This data indicates that MerTK may provide a positive stimulus for PBMCs to recruit them to the tumor, similar to our observations in the TNBC mouse models. Furthermore, a TMA of 119 TNBC clinical samples was stained by multiplex immunofluorescence (mIF) for four immune cell markers: CD8 (T cells), CD20 (B cells), CD68 (macrophages), and CD56 (NK cells). Samples were split into three groups based on tumor MerTK optical density (OD), and the percent of the tumor segment area for each marker was calculated. Little immune infiltrate was detected in the MerTK low group (OD <0.01). However, in the middle and high MerTK expression groups (OD 0.01-0.05 and OD >0.05, respectively), increasing levels of all four analytes are detected in the tumor compartment (**Figures 6F, G**). Together, these data indicate that tumor-bound MerTK contributes to a pro-inflammatory TIME not only in murine models but also in a human *in vitro* model and TNBC clinical samples, suggesting clinical relevance for MerTK as a potential predictive biomarker for tumor temperature and response to ICIs in TNBC.

### 3.6 MerTK and PD-L1 expression are not coupled

**Figure 7:**
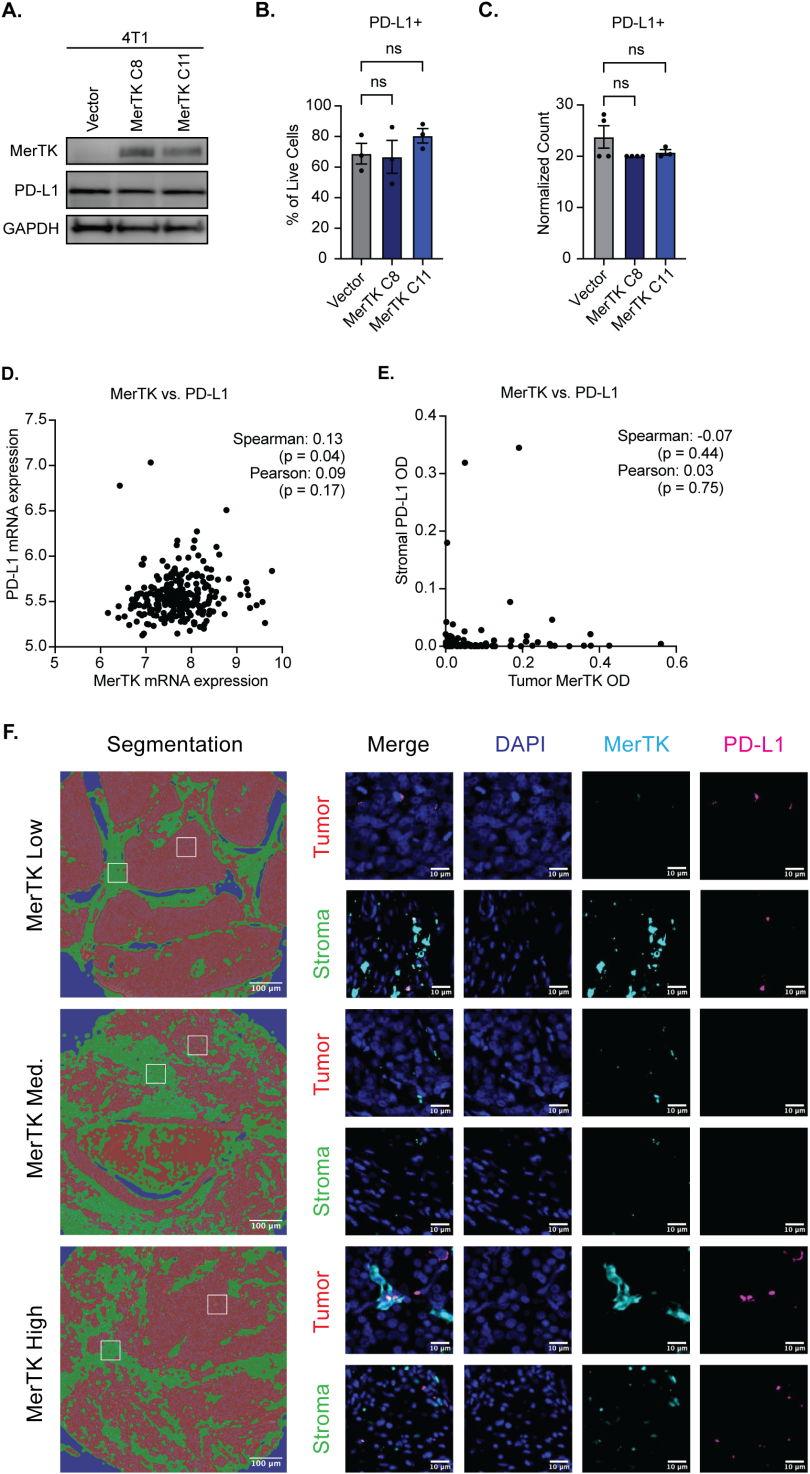
Tumor-bound MerTK is not coupled to PD-L1 expression. **(A)** Whole cell lysate was collected from 4T1 cells overexpressing MerTK and the vector control and was immunoblotted for MerTK, PD-L1, and GAPDH, which was used as a loading control. **(B)** PD-L1 expression was analyzed in 4T1 cells overexpressing MerTK and vector control by flow cytometry. Mean values and SEMs are shown (n=3 per group). **(C)** PD-L1 RNA expression was evaluated in 4T1 cells overexpressing MerTK and vector control by NanoString nCounter. Mean Values and SEMs are shown (n=4 per group). **(D)** Correlation analysis of MerTK and PD-L1 mRNA expression in human TNBC patients. Data was obtained from the cBioportal (n=258 TNBC patients). **(E)** Correlation analysis of tumor-bound MerTK and stromal PD-L1 staining by mIF (n=101 TNBC patients). **(F)** Representative images of tumor-bound MerTK low, medium, and high-expressing patients. DAPI (blue), MerTK (cyan), and PD-L1 (magenta) staining are depicted for both stromal (green) and tumor (red) compartments. ns, not significant.

The *in vivo* data presented thus far suggests a potential role for tumor-bound MerTK as a predictive biomarker for TNBC response to ICIs. Stromal PD-L1 expression is currently the only biomarker used in the clinic to determine TNBC patient eligibility for treatment with pembrolizumab and is only minimally effective in predicting response. Given the remarkable sensitivity to ICIs that we have observed in murine tumors overexpressing MerTK and pro-inflammatory TIME seen in the human KOALA and TMA, we propose tumor-bound MerTK as an additional biomarker to predict patient response to ICI treatment and expand the number of patients who may be eligible to receive life-saving treatment. It is critical to ensure that tumor-bound MerTK and PD-L1 expression are not coupled to propose tumor-bound MerTK as an independent biomarker to PD-L1. In our 4T1 model of TNBC, no change in PD-L1 expression is observed at the protein level by Western blot (**Figure 7A**) or flow cytometry (**Figure 7B**), nor are changes detected at the RNA level by NanoString nCounter analysis (**Figure 7C**) when MerTK is overexpressed. Furthermore, analysis of 258 TNBC tumor samples from the cBioPortal (32–37) revealed no correlation (Spearman’s rank correlation coefficient = 0.13, p-value = 0.04; Pearson = 0.09, p-value = 0.17) between CD274 (PD-L1) and MerTK mRNA (**Figure 7D**). At the protein level, analysis of 100 TNBC clinical samples by mIF revealed no correlation (Spearman’s rank correlation coefficient = -0.07, p-value = 0.44; Pearson = 0.03, p-value = 0.75) between stromal PD-L1 and tumor-bound MerTK optical density (OD) (**Figures 7E, F**). Together, these data indicate that tumor-bound MerTK may serve as a predictor of response to ICI in TNBC, and that further investigation into its use as a biomarker may be warranted.

## 4 Discussion

**Figure 8:**
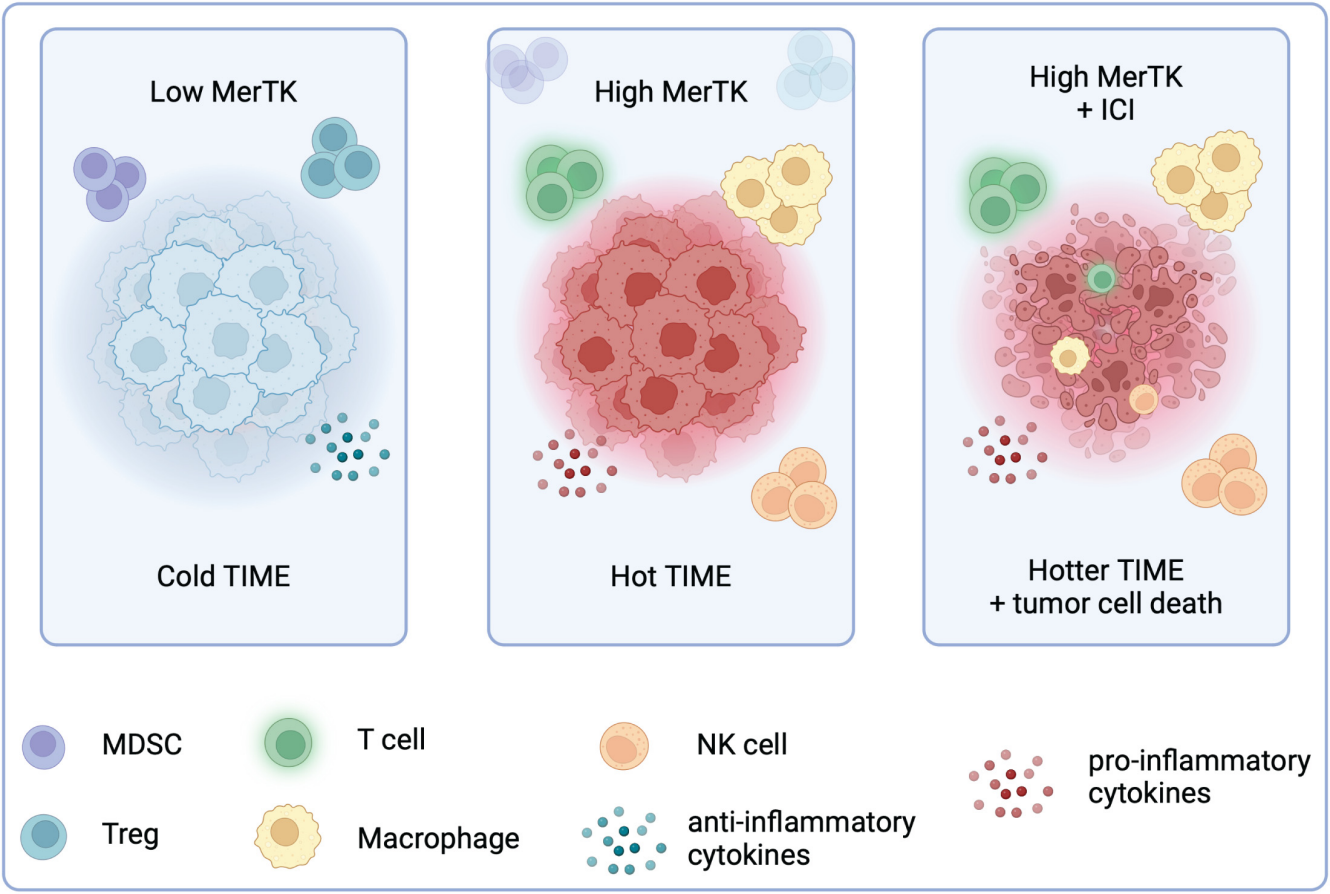
High tumor-bound MerTK expression is associated with a hotter TIME and sensitivity to ICI in TNBC. Created in BioRender. Wheeler, D. (2024) https://BioRender.com/f41u920.

The tumor immune microenvironment is a key determinant of tumor response to immune checkpoint inhibition. Pembrolizumab has shown promise in tumor types with a hotter TIME, including melanoma and NSCLC, but modest efficacy in immune cold tumor types, such as HNSCC and breast cancer (18). Although the TNBC TIME is more inflamed than hormone receptor-positive breast cancer, it is still relatively cold. Pembrolizumab responses remain low in PD-L1 positive patients, revealing a knowledge gap about TNBC sensitivity to ICIs. In this study, we reveal that tumor-bound MerTK uniquely contributes to the development of a hot TIME in TNBC, with high MerTK expression on tumors enhancing their sensitivity to ICI therapy (**Figure 8**).

RTKs have long been implicated in the progression of many solid tumors due to their high expression levels and ability to regulate critical cellular functions, including survival, proliferation, migration, and metabolism (38). Our group has previously reported that in *in vitro* and immunodeficient mouse models of TNBC, MerTK overexpression promotes cell proliferation and contributes to tumor progression and metastasis (9). While the role of the TAM family of RTKs expressed on macrophages and dendritic cells has been studied, the role of tumor-bound MerTK in the tumor immune microenvironment is poorly understood (39).

The current study shows MerTK expression in both human TNBCs (**Figures 3A, B**) and mouse TNBCs (**Figures 3C-G**). While inhibiting MerTK in other cancers (e.g., melanoma, pancreatic, colon cancers, and hepatocellular carcinoma) suppresses pro-tumor immune cells and cytokines (40–42), in TNBC MerTK overexpression uniquely enhances anti-tumor immune infiltration in murine 4T1 tumors (**Figures 1**, **4**). While MerTK seems to play opposing roles across tumor types, this finding exemplifies the ability of signaling pathways to behave differently in different contexts. Though uncommon, genes can exhibit both oncogenic and tumor suppressive functions depending on the environment in which they are expressed. For example, the gene *DYRK1B* is highly expressed in many cancer types where it is involved in arrest of the cell cycle but is also involved in resistance to chemotherapy (43). In glioma, the gene *INPP5F* has been shown to reduce tumor progression, while its expression contributes to metastasis in colon cancer (43, 44). Demirsoy et al. recently reported that tumor growth in the 4T1 model could be abrogated by promoting a hotter TIME when blocking Tyro3, Axl, MerTK, and Met with the small molecule inhibitor sitravatinib and that this blockade enhances the anti-tumor effect of CDK4/6 inhibition (45). In contrast, we demonstrate that MerTK-overexpressing mouse TNBCs are incredibly sensitive to treatment with either αPD-L1 or αCTLA-4 therapeutic antibodies (**Figures 2**, **5**). While the results of our studies differ, given our conclusion that MerTK plays a role in creating a pro-inflammatory TIME in TNBC when expressed on the tumor, we believe it is important to consider localization of MerTK and isolation of MerTK as a single variable when looking at its role in tumor growth and the TIME. While it is likely that stromal expression of MerTK may contribute to a colder TIME and therapeutic resistance, it has also been shown to act as a costimulatory signal for CD8 T cell activation (46). In this study, we also show that MerTK plays a unique role in the human tumor immune microenvironment in *in vitro* models and clinical biospecimens (**Figure 6**), and targeting MerTK as a therapeutic modality in TNBC should be considered with caution.

Immune checkpoint inhibition has become a promising modality for treating a subset of TNBC patients in recent years. In 2020 and 2021, two landmark clinical trials led to FDA approval of the PD-1 inhibitor pembrolizumab for advanced, PD-L1 positive TNBC and early-stage TNBC, respectively (21, 22). While PD-L1 is currently the only biomarker to select patients eligible to receive pembrolizumab, clinical trial data has shown that improvement in pathologic complete response is seen regardless of PD-L1 status (47). Further, evaluation of PD-L1 as a predictive biomarker for response to pembrolizumab across tumor types revealed that PD-L1 expression was only predictive in 28.8% of FDA approvals, indicating the imprecise nature of utilizing PD-L1 as a predictive biomarker (48). Additionally, there is no predictive biomarker for patient response to pembrolizumab in the early-stage TNBC setting, and patients are at high risk of immune-related adverse effects (49). Additional biomarkers for patient response must be brought to the clinic to expand inclusion criteria in the late-stage setting and reduce toxicity by treating only patients predicted to respond in the early-stage setting. In addition to predicting response to pembrolizumab, MerTK may also serve as a predictive biomarker for other immune checkpoint inhibitors, such as CTLA-4, given the durable response observed in our murine model of TNBC (**Figures 2**, **5**). Finally, while studies have shown that stromal MerTK and PD-L1 expression are coupled in different cell types (50–52), we report that expression of tumor-bound MerTK is not coupled to stromal PD-L1 expression in TNBC (**Figure 7**). Thus, our preclinical findings identify tumor-bound MerTK as a promising candidate for a biomarker, independent of PD-L1 expression, to predict TNBC response to immune checkpoint therapy.

## Data Availability

The raw data supporting the conclusions of this article will be made available by the authors, without undue reservation.
