# Supplementary material for: Tumor-associated MerTK promotes a pro-inflammatory microenvironment and enhances immune checkpoint inhibitor response in triple-negative breast cancer
Source: Front Oncol. 2025 May 5;15:1579214. doi: 10.3389/fonc.2025.1579214 (PMC12086072; doi:10.3389/fonc.2025.1579214)
Supplement: Supplementary file 1 [file DataSheet1.docx]

Supplementary Material


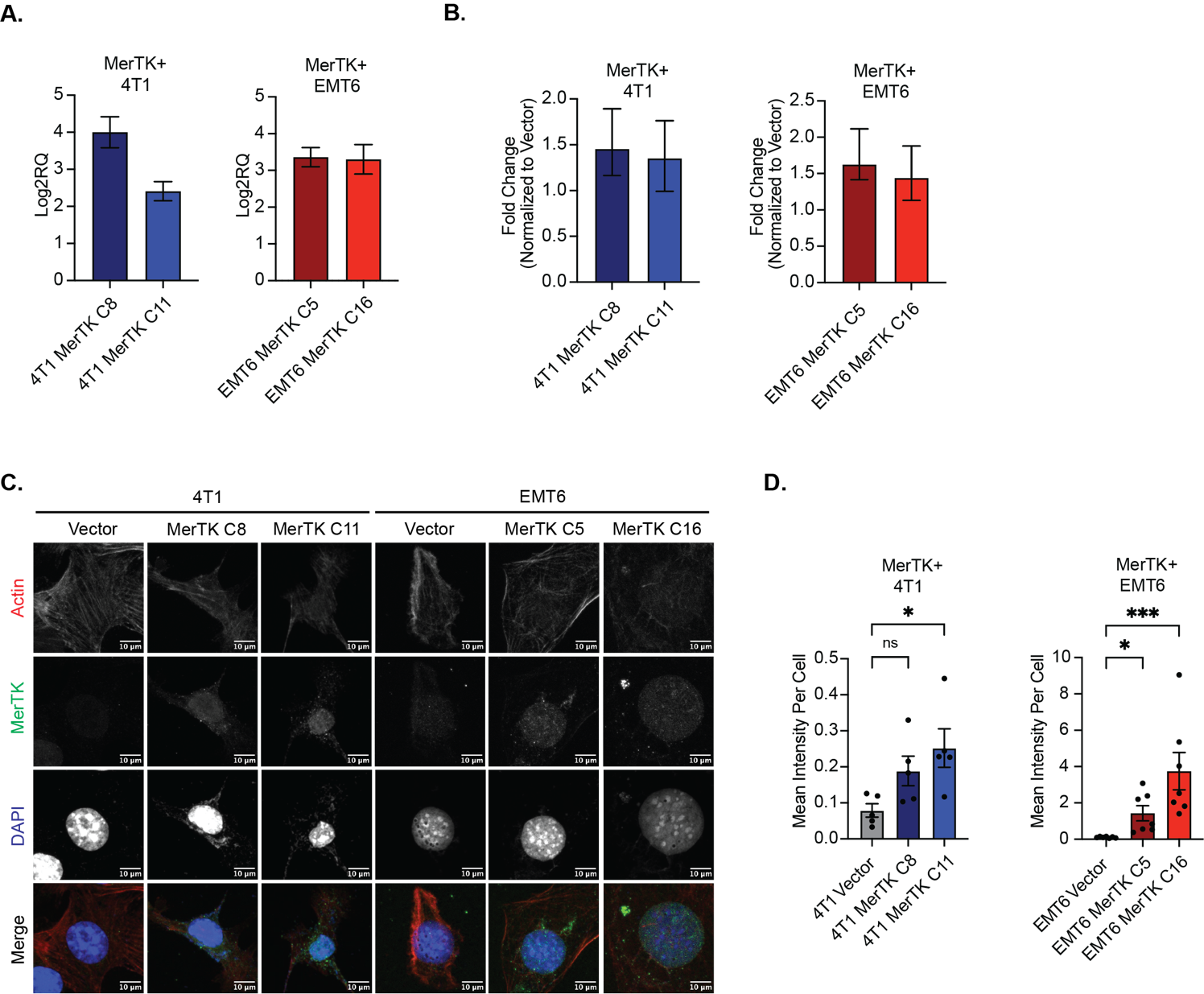


**Supplementary Figure S1.** MerTK overexpression in two murine models of TNBC. A) RNA was isolated from 4T1 MerTK and EMT6 MerTK overexpressing clones and their vector controls and subjected to qPCR analysis for MerTK gene expression. Mean values and SEMs are shown (n=3 per group). B) MerTK expression in 4T1 MerTK and EMT6 MerTK overexpressing clones and their vector controls were analyzed by flow cytometry. Fold changes relative to 4T1 Vector control are shown (n=6 per group). C) 4T1 MerTK and EMT6 MerTK overexpressing clones and their vector controls were analyzed by confocal microscopy for MerTK expression. Representative images taken at 100x magnification depict actin (red), MerTK (green), and DAPI (blue) staining.D) Quantification of MerTK expression using FIJI V2.14.0/1.54f. Mean values and SEMs are shown (n=5-7 images taken at 40x magnification per group). EMT6 cells were stimulated with 1ug/mL dox for 16 hours prior to experimentation. *P<0.05; ***P<0.001; ns = not significant.


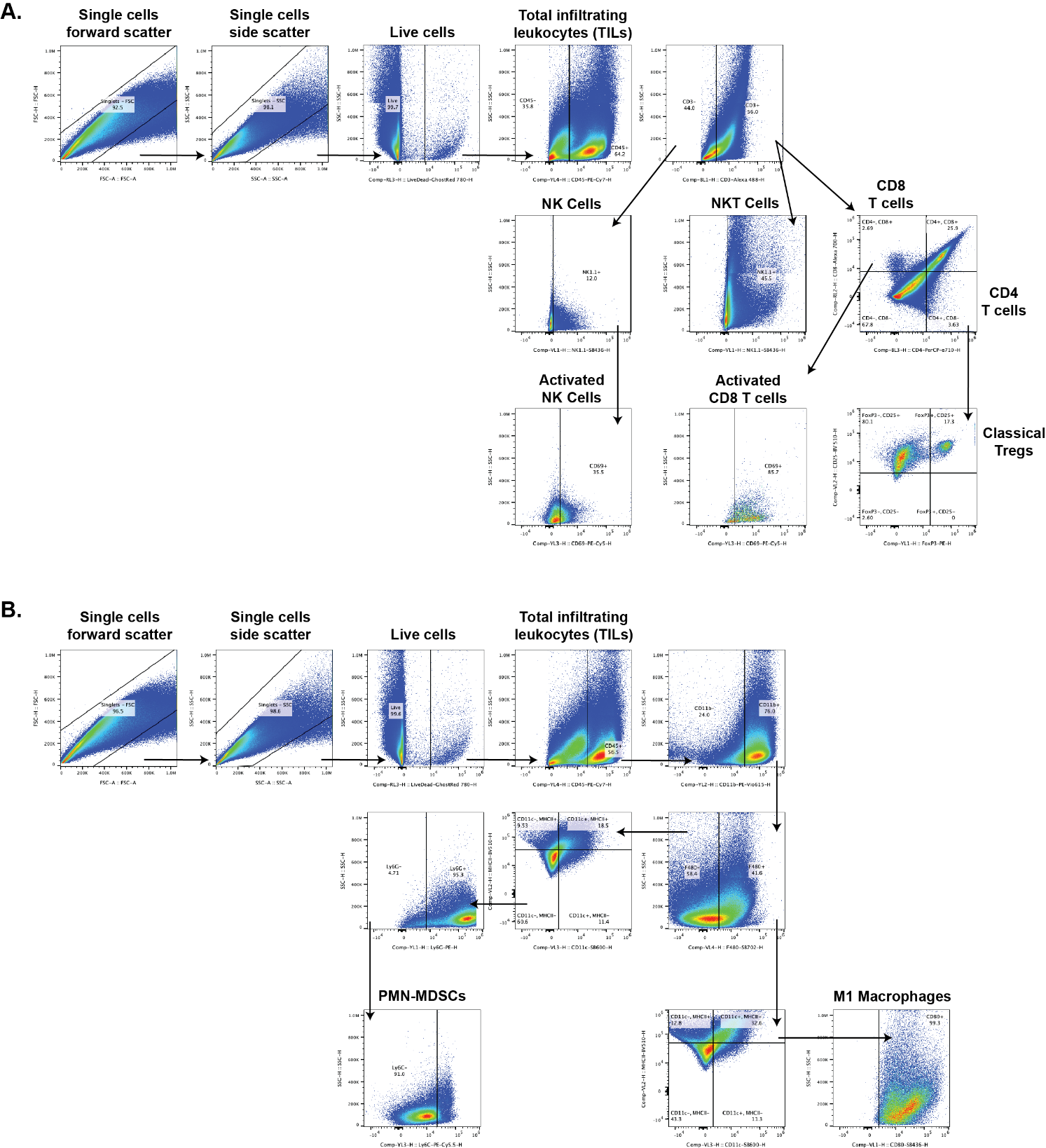


**Supplementary Figure S2.** Gating strategies for flow cytometry analysis of cells of A) lymphoid or B) myeloid lineage.


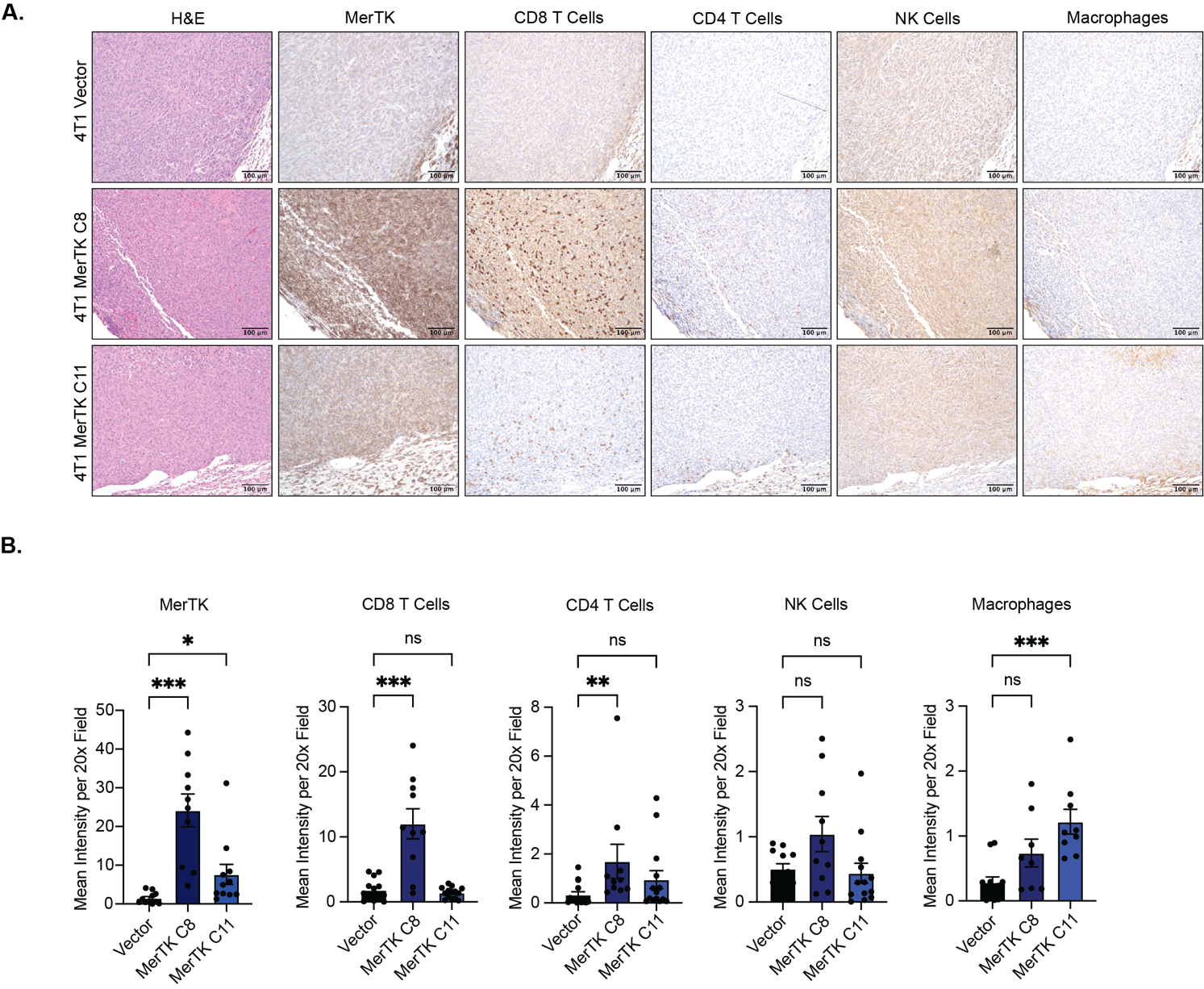


**Supplementary Figure S3.** MerTK expression promotes anti-tumor immune infiltration. A) Tumors were harvested, processed, and stained using IHC. Representative images are shown at 20x magnification. B) Tumor immune infiltrate by IHC was quantified using FIJI V2.14.0/1.54f. Mean values and SEMs are shown (n=8-14 tumors per group). **P*<0.05; ** *P*< 0.01; *** *P*< 0.001; ns = not significant.


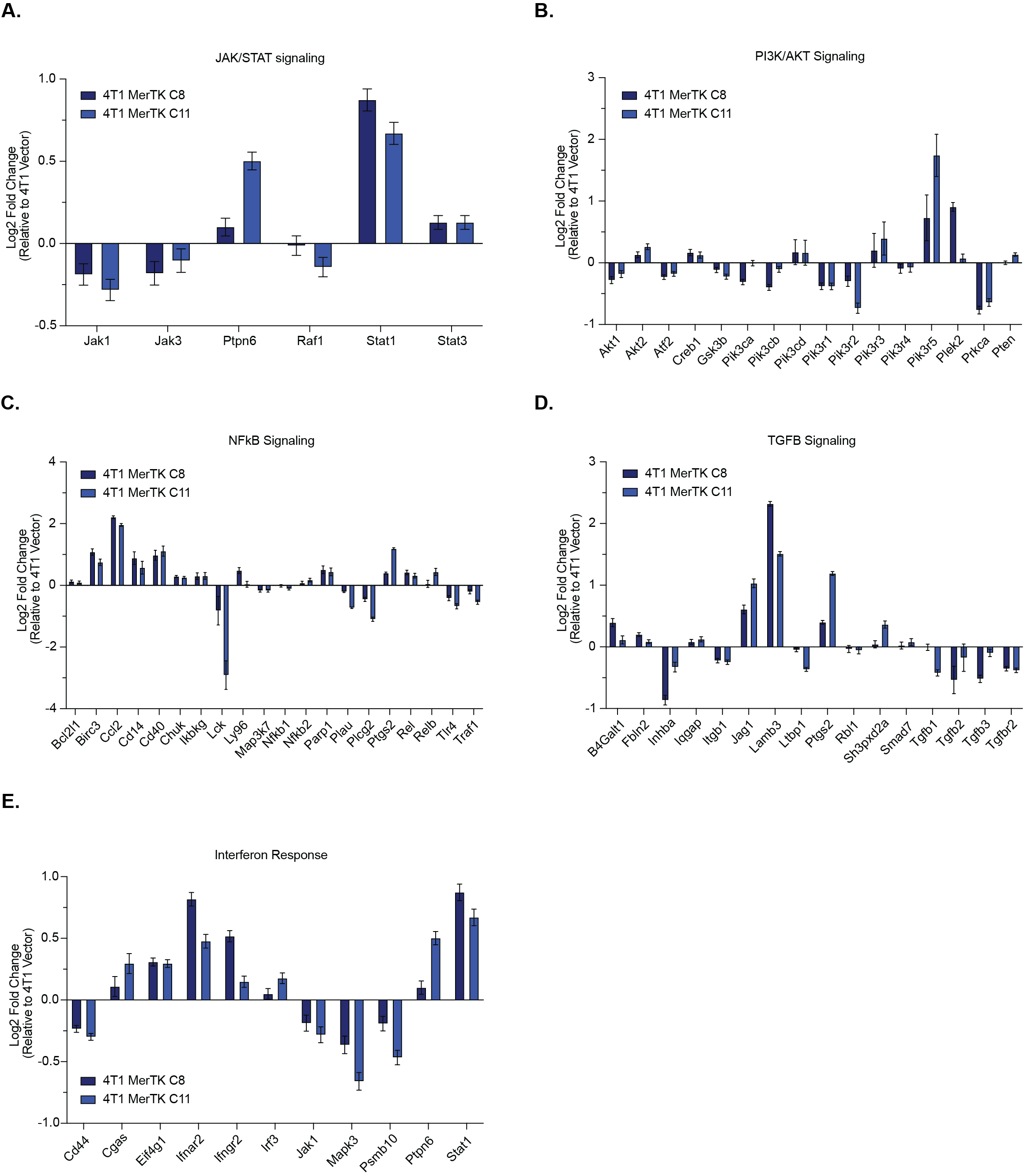


**Supplementary Figure S4.** MerTK expression alters immunological signaling pathways A-D) RNA was isolated from cultured cells and subjected to NanoString nCounter analysis. Genes involved in A) JAK/STAT, B) PI3K/AKT, C) NFκB, D) TGFβ, and E) interferon signaling are differentially expressed in MerTK-overexpressing cells. Log2 fold change relative to 4T1 Vector and SEMs are shown (n=4 tumors per group).


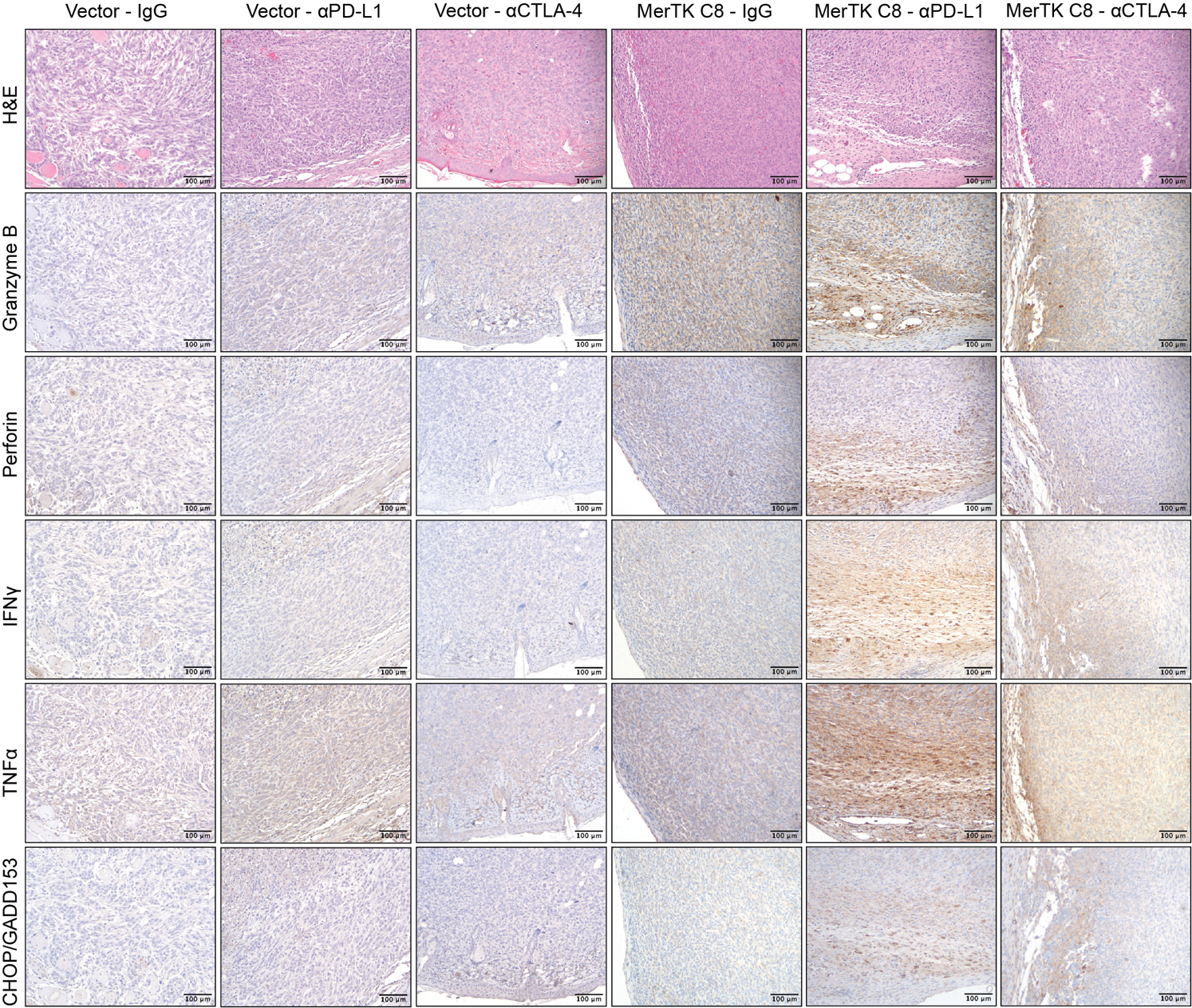
w

**Supplementary Figure S5.** Treatment with ICI in MerTK-overexpressing tumors results in increased tumor cell killing. Tumors were harvested, processed, and stained using IHC. Representative images are shown at 20x magnification.


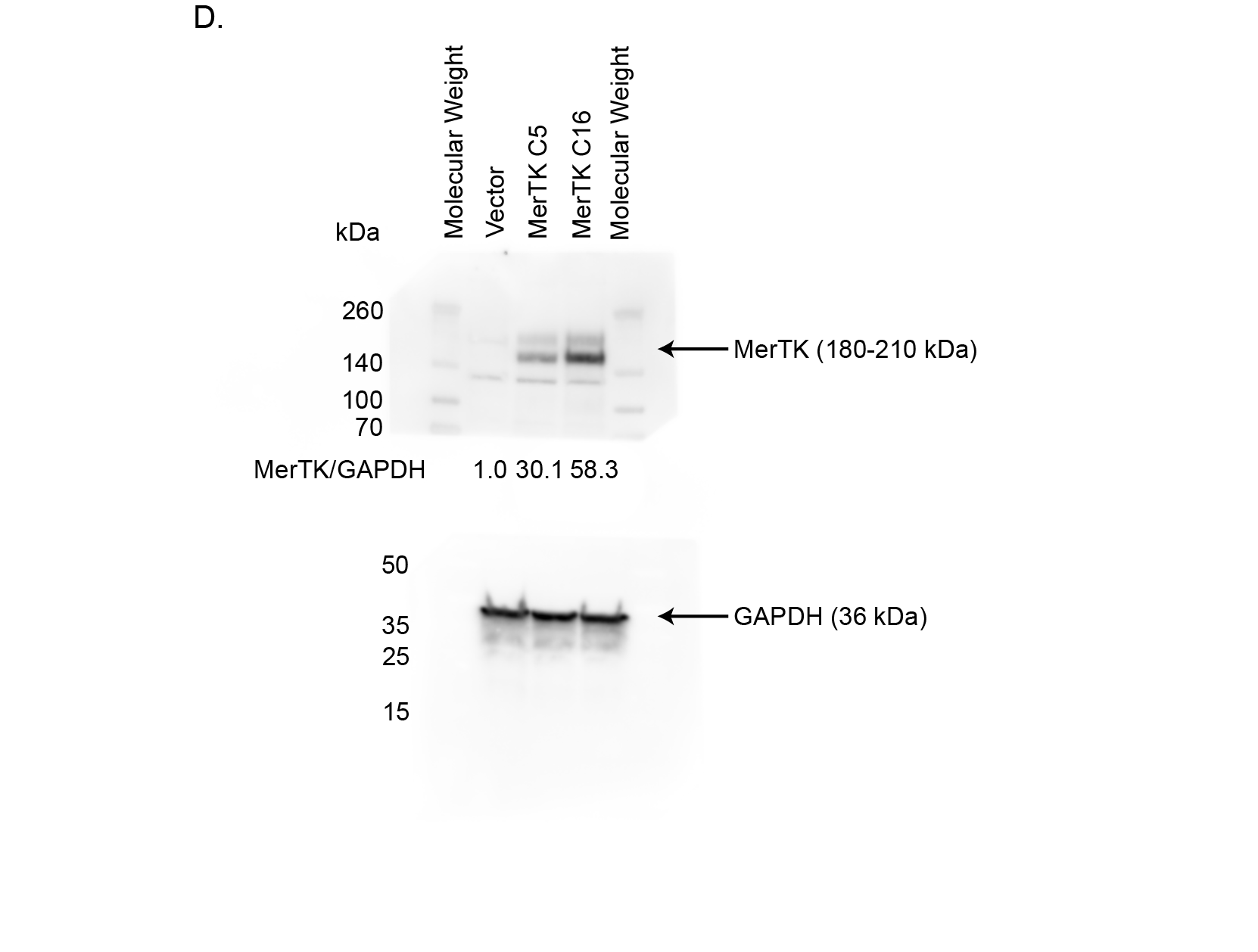

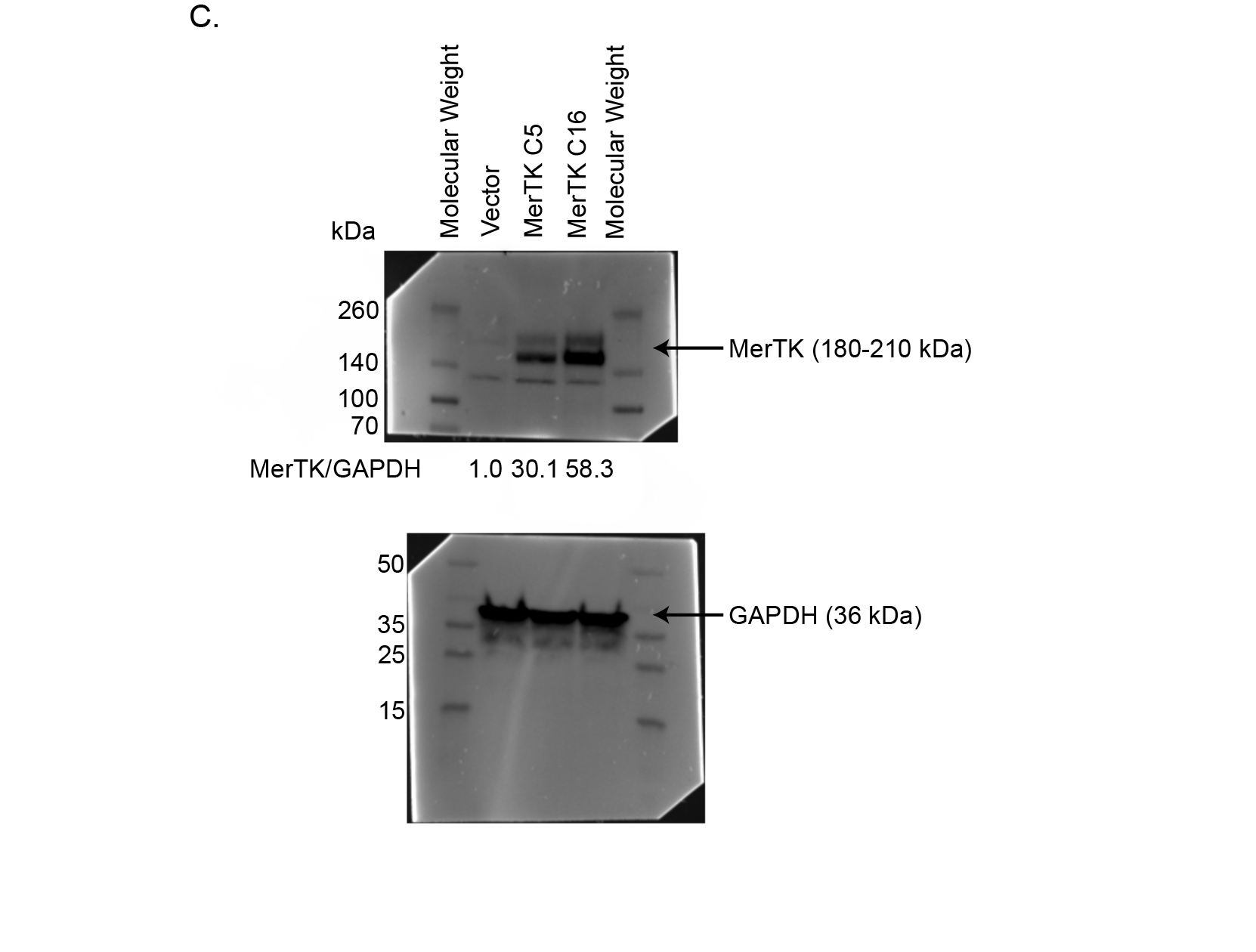

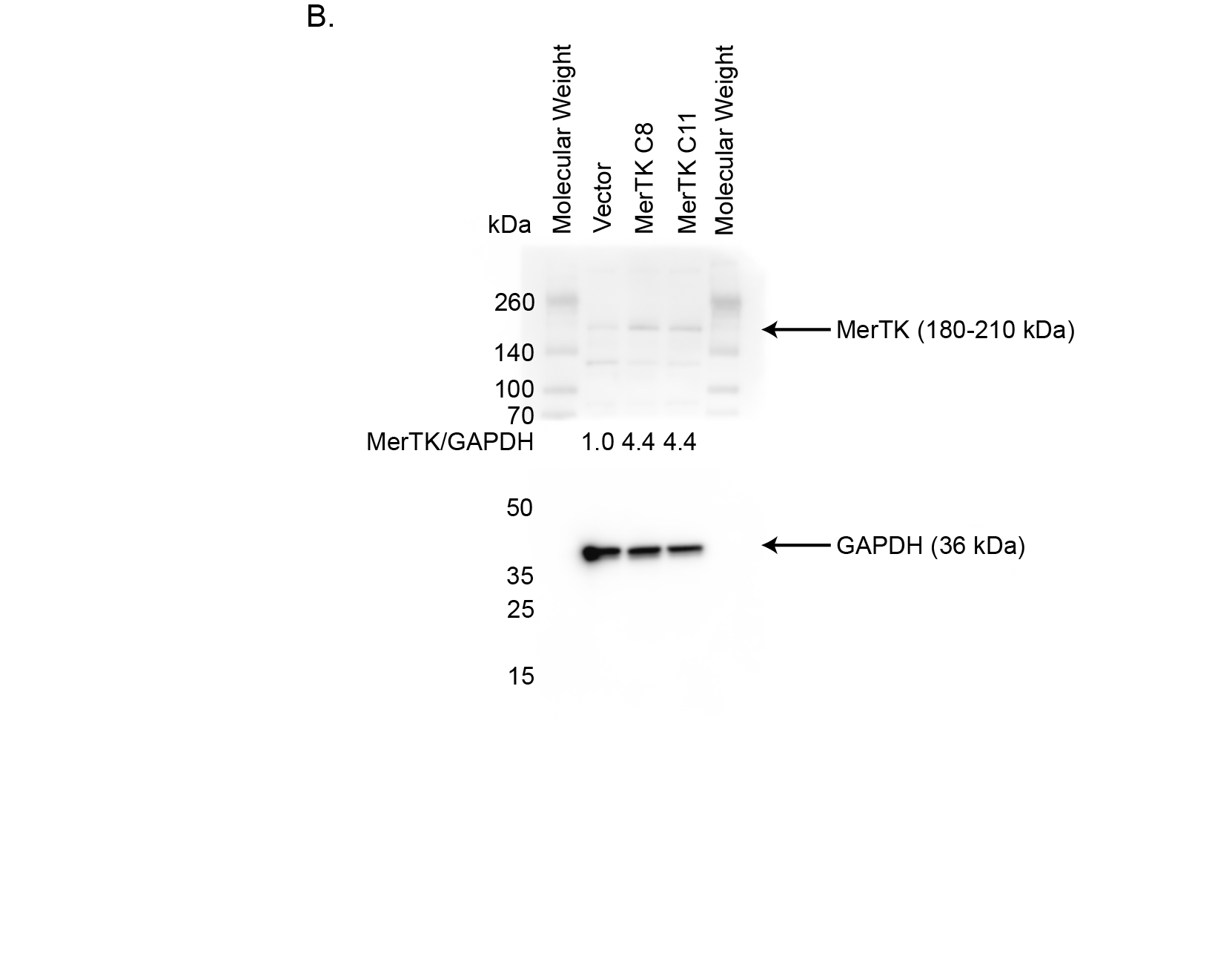

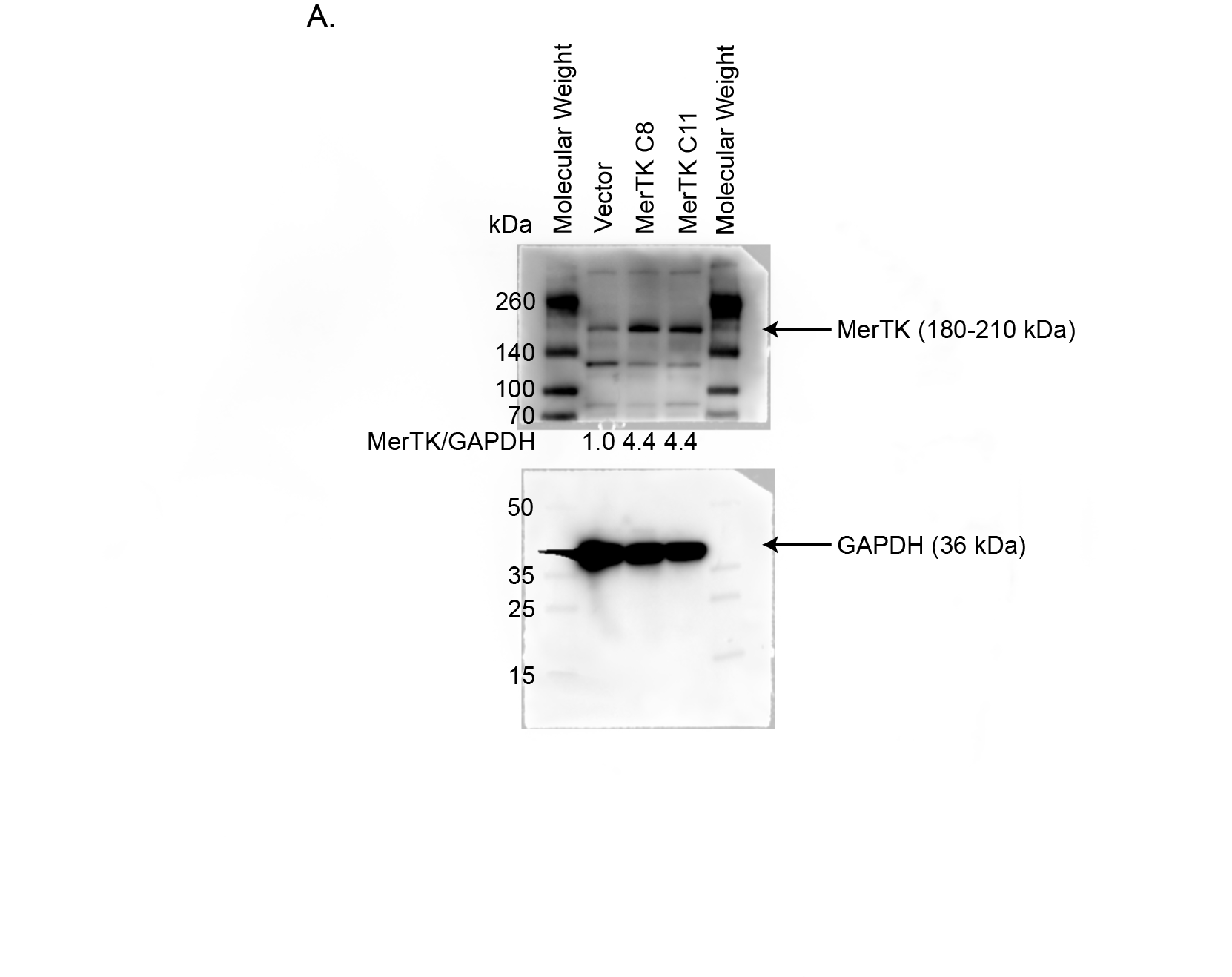


**Supplementary Figure S6**. Uncropped western Blot images for Figure 1C. A) 4T1 MerTK and GAPDH blots with marker overlay (long exposure). B) 4T1 MerTK and GAPDH blots (short exposure). C) EMT6 MerTK and GAPDH blots with marker overlay (long exposure). D). EMT6 MerTK and GAPDH blots (short exposure).


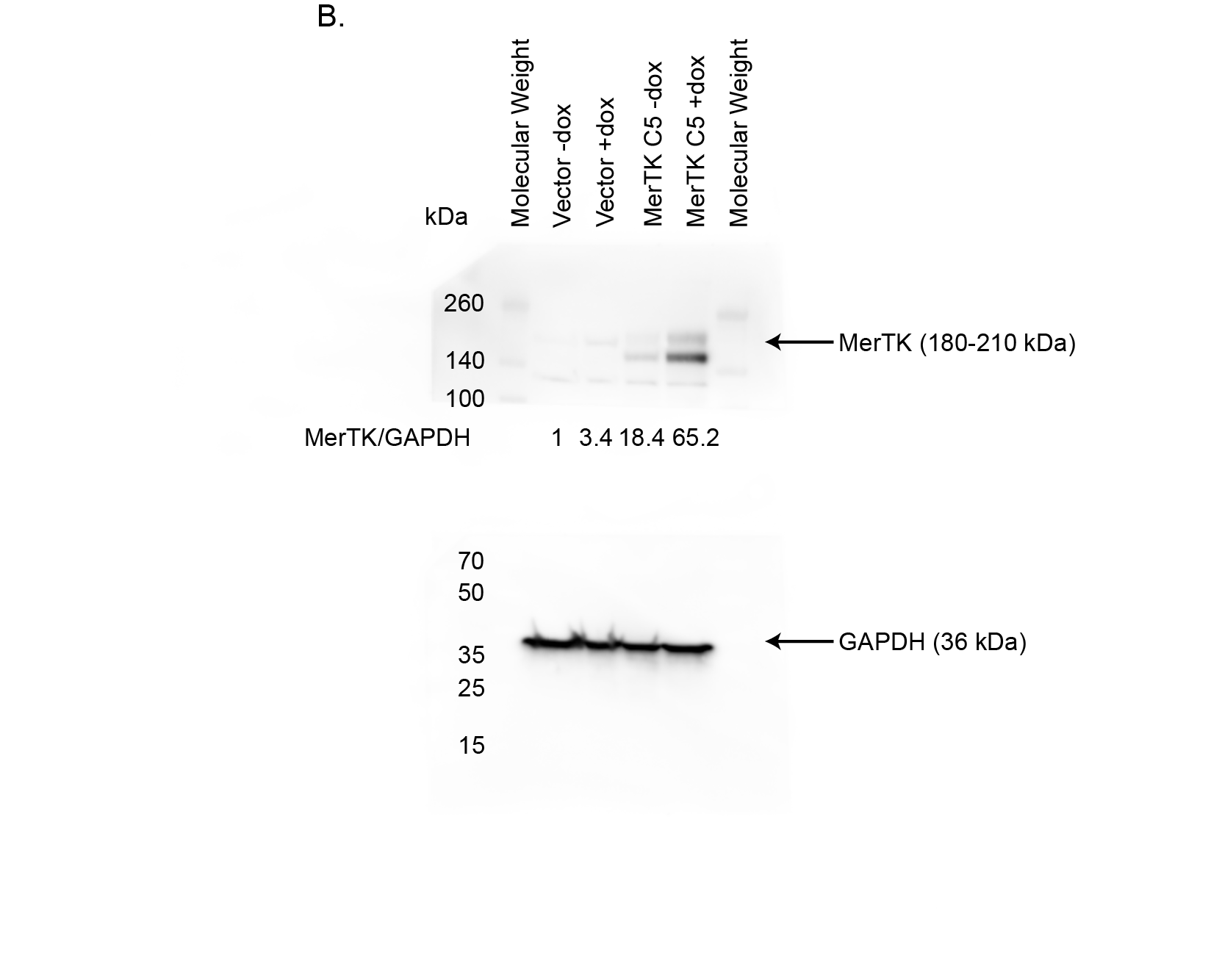

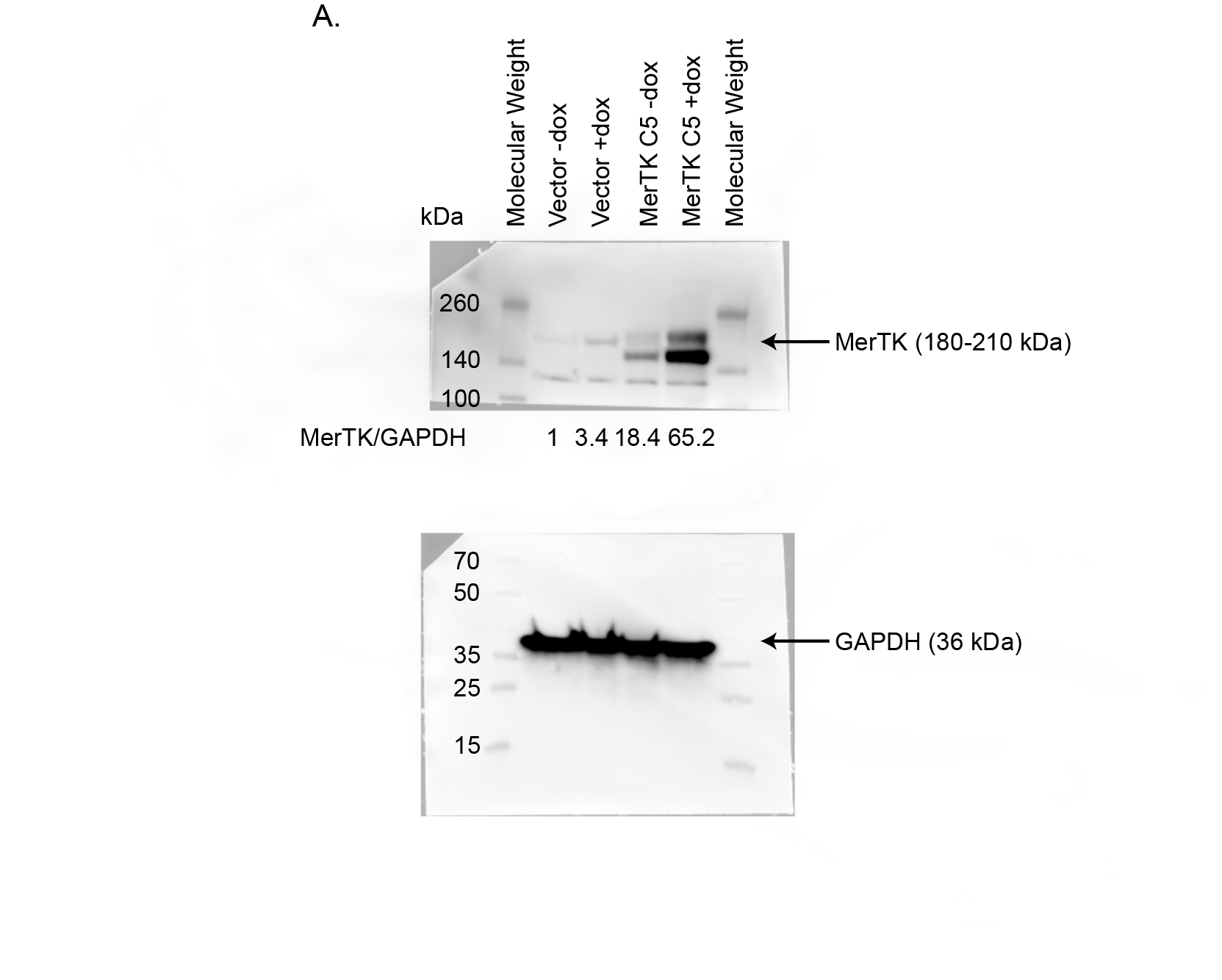


**Supplementary Figure S7.** Uncropped western Blot images for Figure 3A. A) MerTK and GAPDH blots with Marker overlay (long exposure). B) MerTK and GAPDH blots (short exposure).


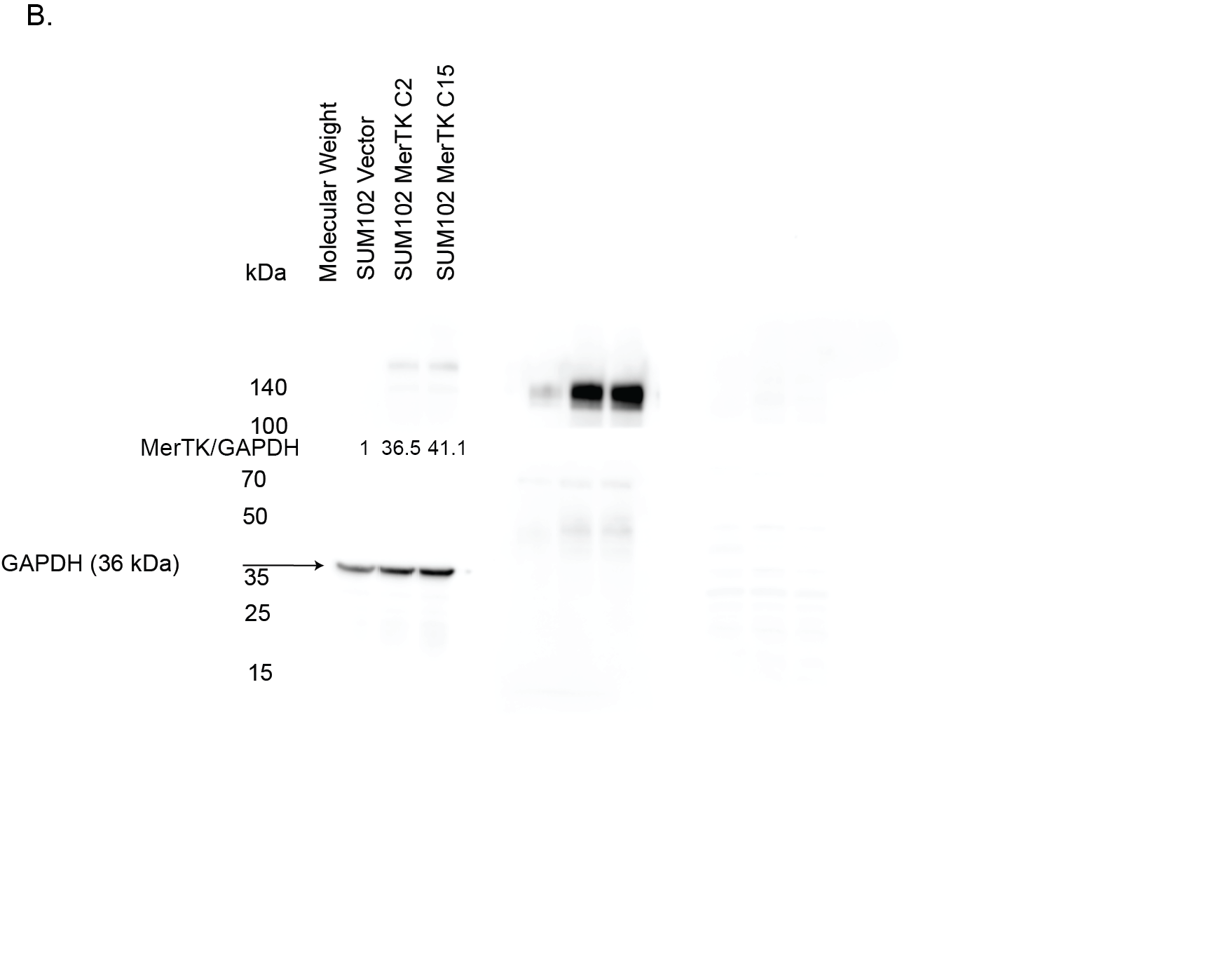

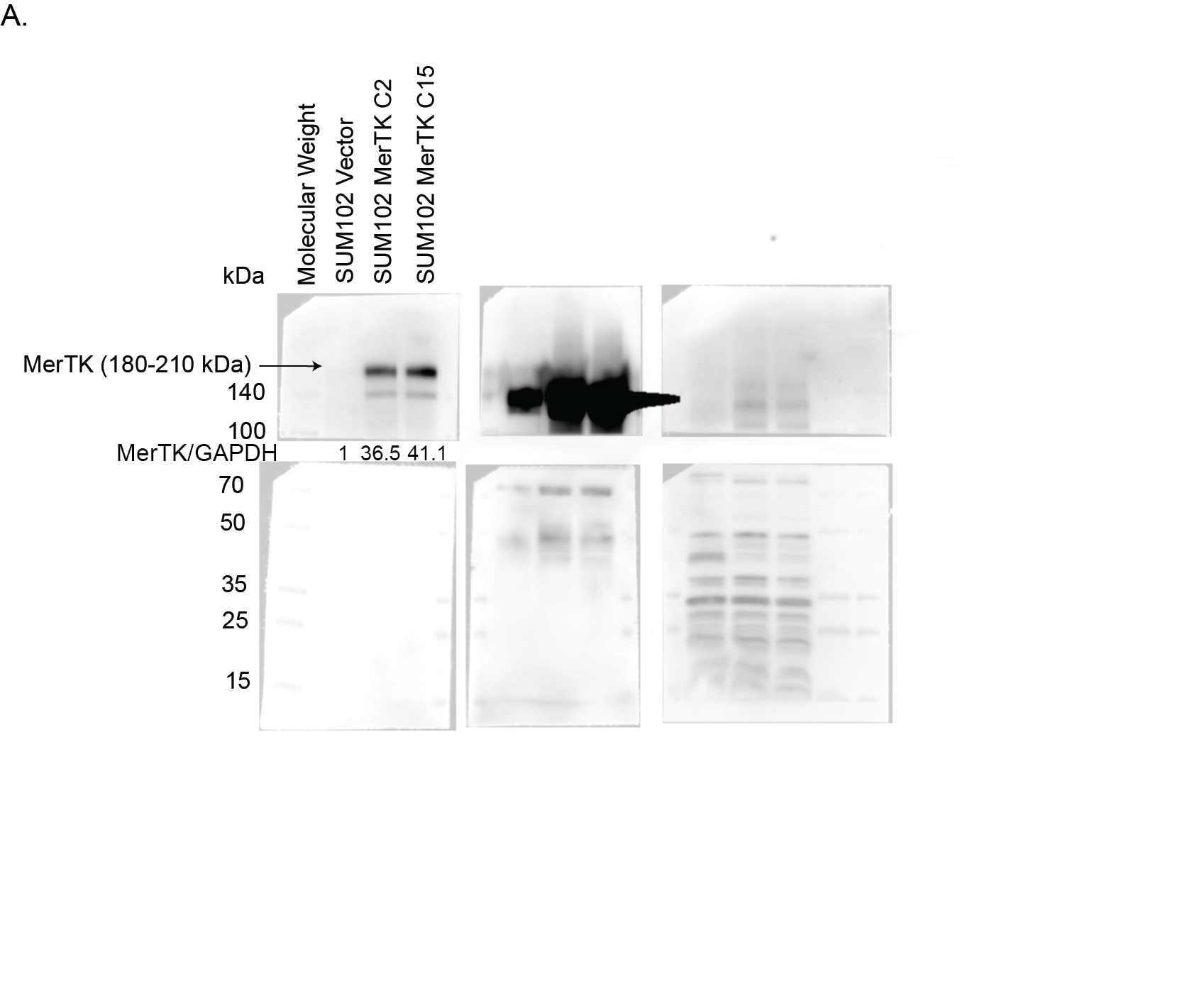


**Supplementary Figure S8.** Uncropped western Blot images for Figure 6A. A) MerTK and GAPDH blots with Marker overlay (long exposure). B) MerTK and GAPDH blots (short exposure).


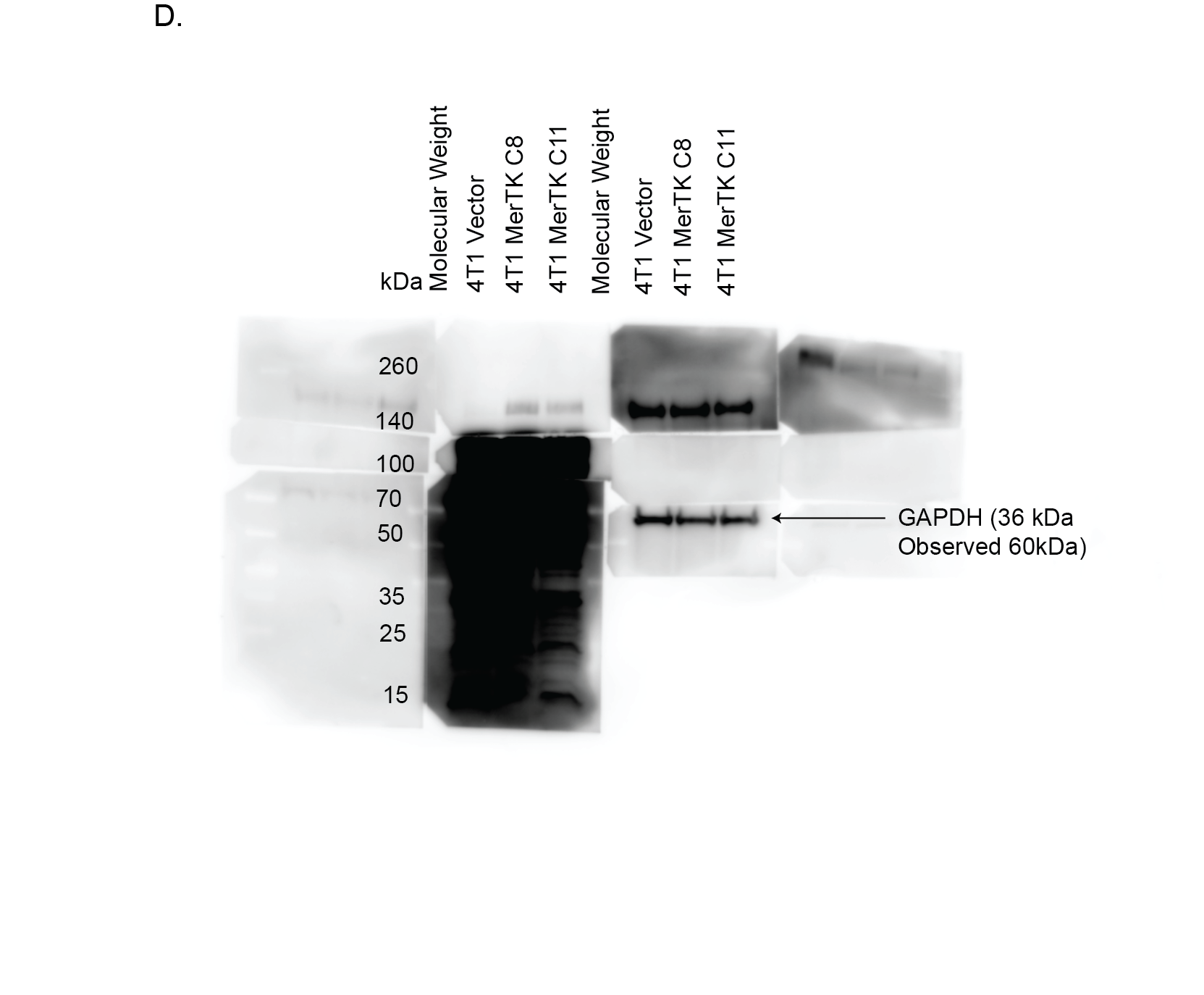

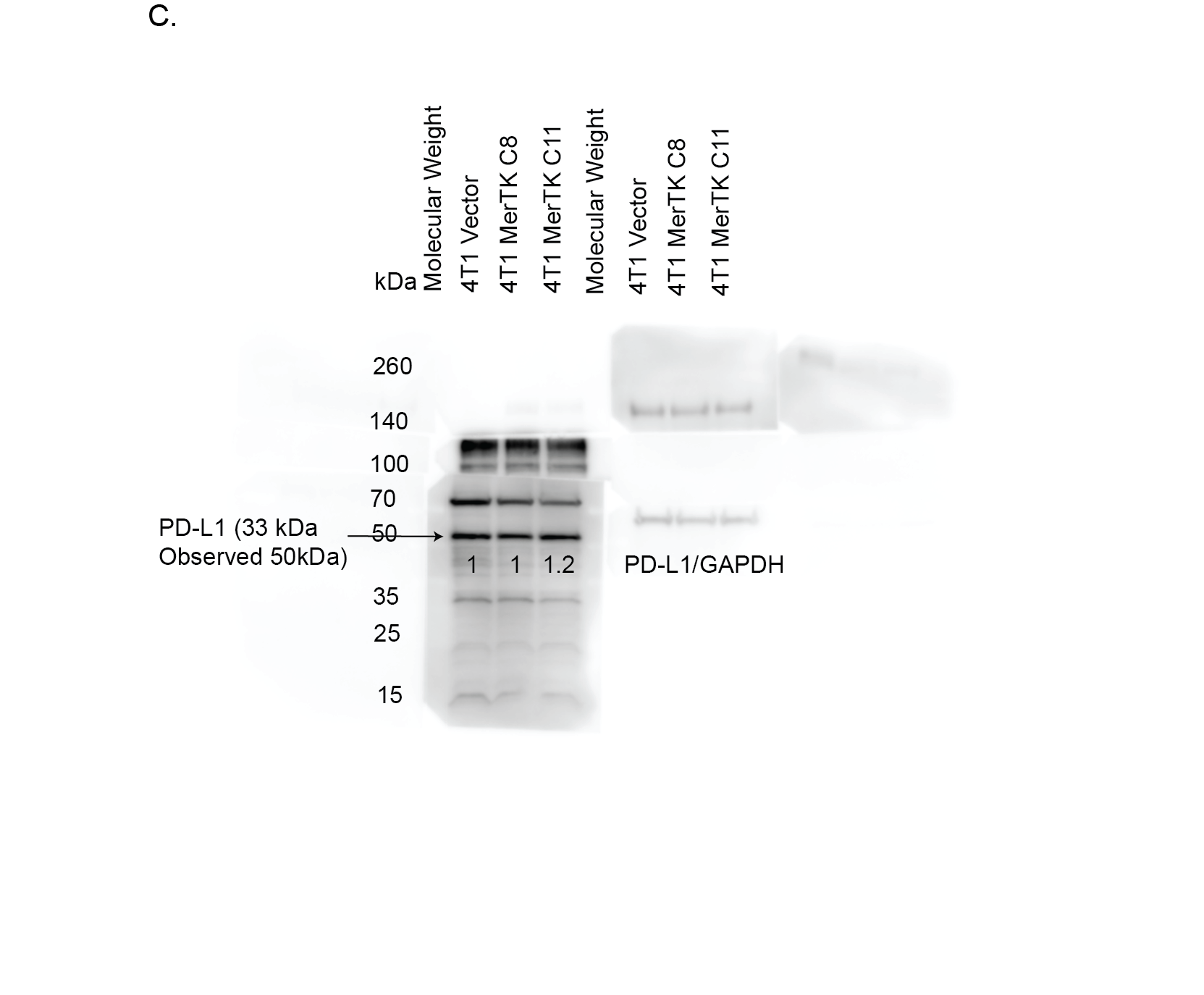

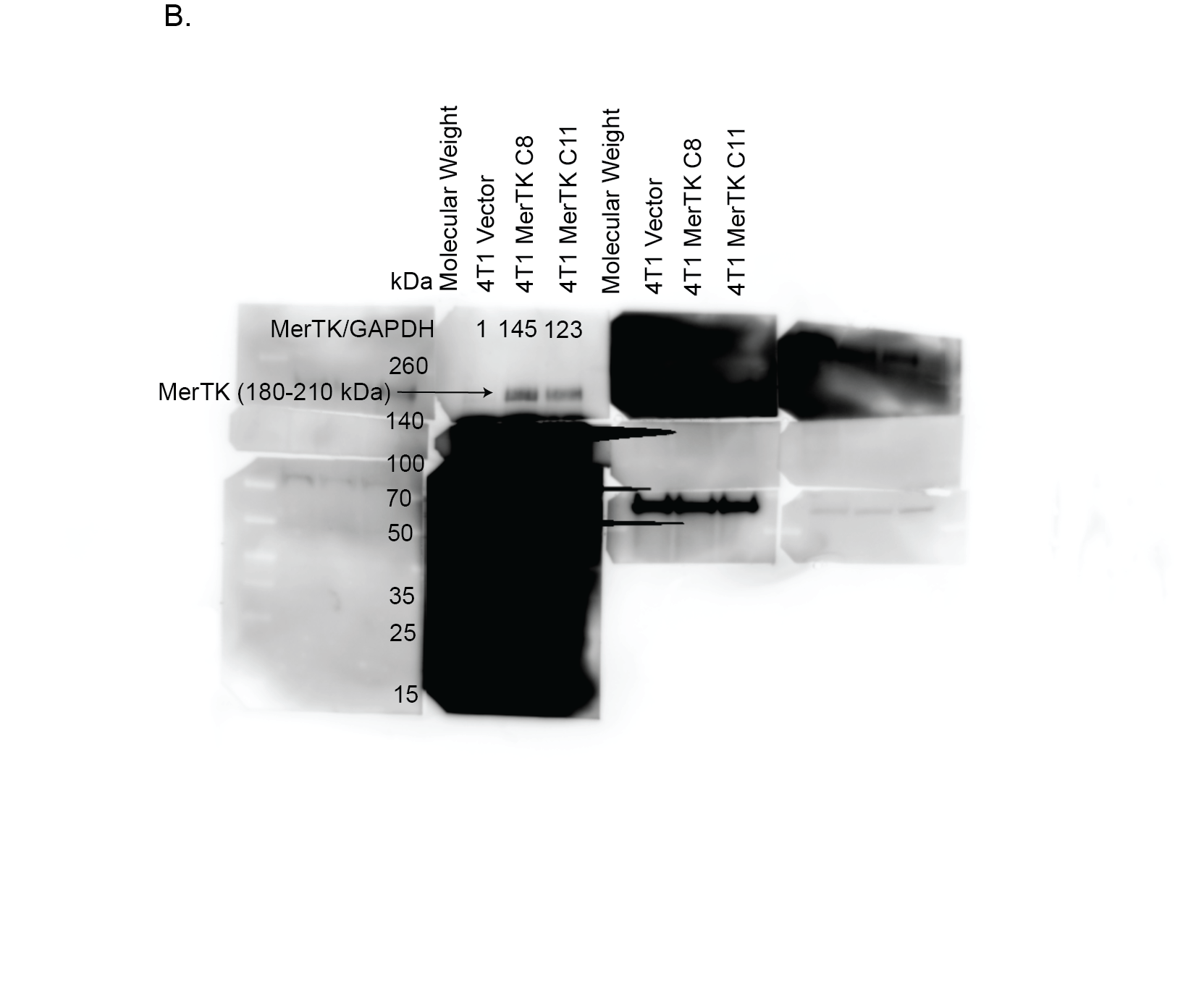

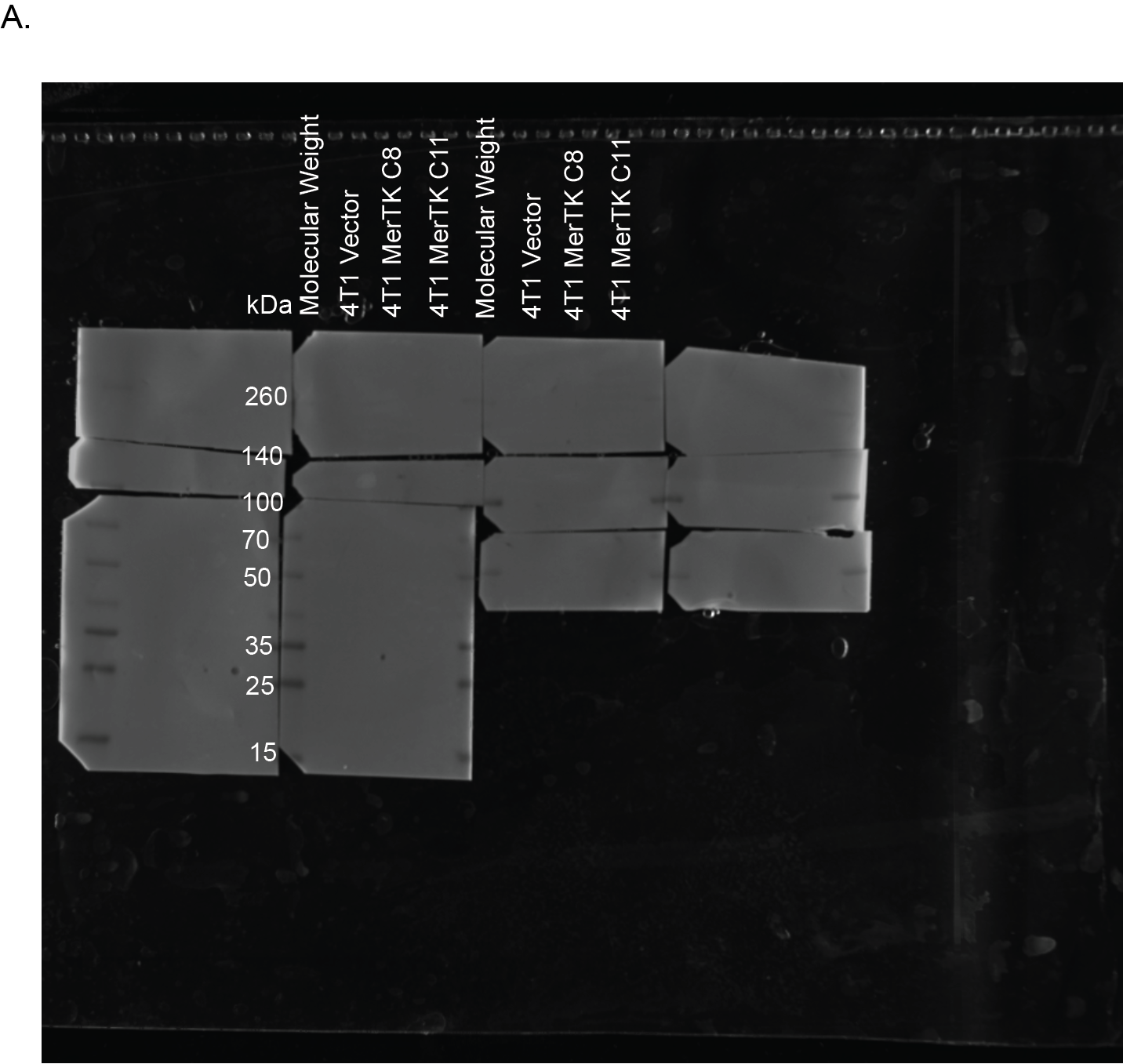


|  |
| --- |

**Supplementary Figure S9.** Uncropped western Blot images for figure 7A. A) Marker. B) MerTK blot (long exposure). C) PD-L1 blot (short exposure). C) GAPDH blot (medium exposure).

**Supplementary Table S1.** IHC antibodies

| **Antigen** | **Vendor** | **Catalog Number** | **Dilution** |
| --- | --- | --- | --- |
| CD8 | Invitrogen | 14-0808-82 | 1:500 |
| CD4 | Invitrogen | 14-9766-82 | 1:500 |
| NKp46 | Thermo Fisher Scientific, Waltham, MA, USA | PA5-102860 | 1:200 |
| F4/80 | Invitrogen | 14-4801-82 | 1:200 |
| FoxP3 | Invitrogen | 14-5773-826 | 1:200 |
| Granzyme B | Santa Cruz Biotechnolgoies Dallas, TX, USA | sc-8022 | 1:200 |
| Perforin | Santa Cruz Biotechnolgoies Dallas, TX, USA | sc-136994 | 1:200 |
| IFN-γ | Santa Cruz Biotechnolgoies Dallas, TX, USA | sc-74108 | 1:200 |
| MerTK | Abcam | Ab184086 | 1:500 |
| Ki67 | Cell Signaling Technologies Danvers, MA, USA | 12202 | 1:400 |

**Supplementary Table S2.** Flow cytometry antibodies

| **Antigen** | **Fluorescent Marker** | **Vendor** |
| --- | --- | --- |
| CD11b | PE-Vio615 | Miltenyi Biotech  Bergisch Gladbach, Germany |
| CD11c | SuperBright 600 | Thermo Fisher Scientific  Waltham, MA, USA |
| CD170 (Siglec F) | BB515 | BD  Franklin Lakes, NJ, USA |
| CD25 | BrilliantViolet 510 | Biolegend  San Diego, CA, USA |
| CD3 | AlexaFluor 488 | Biolegend  San Diego, CA, USA |
| CD4 | PerCP-eFluor 710 | Thermo Fisher Scientific  Waltham, MA, USA |
| CD45 | PE-Cy7 | Biolegend  San Diego, CA, USA |
| CD80 | SuperBright 436 | Thermo Fisher Scientific  Waltham, MA, USA |
| CD8a | AlexaFluor 700 | Biolegend  San Diego, CA, USA |
| F4/80 | SuperBright 702 | Thermo Fisher Scientific  Waltham, MA, USA |
| Live/Dead | GhostRed 780 | Tonbo Biosciences  San Diego, CA, USA |
| Ly6C | PE-Cy5.5 | Elabscience  Wuhan, China |
| Ly6G | PE | Tonbo Biosciences  San Diego, CA, USA |
| MHC-II (I-Ad) | BrilliantViolet 510 | BD  Franklin Lakes, NJ, USA |
| NKp46 | SuperBright 436 | Thermo Fisher Scientific  Waltham, MA, USA |
| Perforin | APC | Biolegend  San Diego, CA, USA |
| MerTK | PerCP-eFluor 710 | Thermo Fisher Scientific  Waltham, MA, USA |
| MerTK | AlexaFluor 647 | Santa Cruz Biotechnologies  Dallas, TX, USA |
| PD-L1 | PE-Cy7 | Tonbo Biosciences  San Diego, CA, USA |

**Supplementary Table S3.** Flow cytometry gating paths

| **Cell Type** | **Gating Path** |
| --- | --- |
| CD4 T Cells | FSC Singlets/SSC Singlets/GR780-/CD45+/CD3+/CD4+, CD8- |
| CD8 T Cells | FSC Singlets/SSC Singlets/GR780-/ CD45+/CD3+/CD4-, CD8+ |
| Activated CD8 T Cells | FSC Singlets/SSC Singlets/GR780-/ CD45+/CD3+/CD4-, CD8+, CD69+ |
| Classical Tregs | FSC Singlets/SSC Singlets/GR780-/ CD45+/CD3+/CD4+, CD8-/FoxP3+, CD25+ |
| NKT Cells | FSC Singlets/SSC Singlets/GR780-/ CD45+/CD3+/NKp46+ |
| NK Cells | FSC Singlets/SSC Singlets/GR780-/ CD45+/CD3-/NKp46+ |
| Activated NK Cells | FSC Singlets/SSC Singlets/GR780-/ CD45+/CD3-/NKp46+/CD69+ |
| M1 Macrophages | FSC Singlets/SSC Singlets/GR780-/ CD45+/CD11b+/F480+/CD11c+,MHCII+/CD80+ |
| M2 Macrophages | FSC Singlets/SSC Singlets/GR780-/ CD45+/CD11b+/F480+/CD11c-, MHCII+ |
| M-MDSCs | FSC Singlets/SSC Singlets/GR780-/ CD45+/CD11b+/F480+/CD11c-, MHCII-/CD170-/Ly6G-/Ly6C+ |
| PMN-MDSCs | FSC Singlets/SSC Singlets/GR780-/CD45+/CD11b+/F480-/CD11c-, MHCII-/Ly6G+/Ly6C- |
| MerTK positive cells | FSC Singlets/SSC Singlets/GR780-/MerTK+ |
| PD-L1 positive cells | FSC Singlets/SSC Singlets/GR780-/PDL1+ |
| TILs – Myeloid, TILs - Lymphoid | FSC Singlets/SSC Singlets/Gr780-/CD45+ |

**Supplementary Table S4.** mIF antibodies – immune profiling

| **Antigen** | **Vendor** | **Catalog Number** | **Dilution** | **Opal** | **Color** |
| --- | --- | --- | --- | --- | --- |
| MerTK | Abcam  Cambridge, UK | AB52968 | 1:100 | 520 | Green |
| CD8 | Ventana Medical Systems  Santa Clara, CA | 790-440 | RTU | 540 | Yellow |
| CD68 | Ventana Medical Systems  Santa Clara, CA | 790-2931 | RTU | 570 | Magenta |
| NCAM1 (CD56) | Cell Signaling Technologies  Danvers, MA | 99746 | 1:50 | 620 | Cyan |
| CD20 | Ventana Medical Systems  Santa Clara, CA | 790-4431 | RTU | 650 | Pink |
| Pan-Cytokeratin | Abcam  Cambridge, UK | AB234297 | 1:50 | 690 | Red |

**Supplementary Table S5.** mIF antibodies – MerTK vs. PD-L1 correlation

| **Antigen** | **Vendor** | **Catalog Number** | **Dilution** | **Opal** | **Color** |
| --- | --- | --- | --- | --- | --- |
| PD-L1 | Cell Signaling Technologies  Danvers, MA | 13684 | 1:50 | 520 | Magenta |
| MerTK | Abcam  Cambridge, UK | ab52968 | 1:100 | 570 | Green |
| Pan-Cytokeratin | Abcam  Cambridge, UK | ab234297 | 1:100 | 650 | Red- |
